# Addressing the inverse care law in Scottish general practice: systematic scoping review

**DOI:** 10.3399/BJGP.2024.0622

**Published:** 2025-07-14

**Authors:** James Bogie, Meike van Dijk, Cara Bezzina, Carey Lunan, David Henderson, Stewart W. Mercer, David N Blane

**Affiliations:** 1 School of Health & Wellbeing, University of Glasgow, Glasgow, Scotland, UK; 2 Faculty of Medical Sciences, Radboud University, Nijmegen, the Netherlands; 3 College of Medicine and Veterinary Medicine, University of Edinburgh, Edinburgh, UK; 4 Centre for Population Health Sciences, Usher Institute, University of Edinburgh, Edinburgh, UK

**Keywords:** general practice, health disparities, health inequities, primary health care, social deprivation, workforce

## Abstract

**Background:**

Recent evidence demonstrates persistence of the inverse care law (ICL) in general practice in England. Although the ICL was well-described in Scotland 20 years ago, progress in interventions since then is unclear.

**Aim:**

To review national and local interventions that aimed to specifically address the ICL in Scottish general practice since 2000.

**Design and setting:**

This was a systematic scoping review set in Scotland.

**Method:**

Embase, Web of Science, PubMed, CINAHL, Cochrane and BASE from 2000 to February 2024 were searched. A systematic grey literature search of government, NHS and third-sector websites was also performed. All papers were double screened for inclusion. Both quantitative and qualitative studies were included and quality was assessed using the Joanna Briggs Institute tools.

**Results:**

Out of 13 089 results, 67 papers reporting on 20 interventions were included. Interventions to improve general practice in deprived areas were categorised as: (a) enhancing patients’ financial or social support, (b) targeting specific health conditions, (c) targeting specific groups, and (d) enhancing generalist health care. Six interventions accounted for 66% (44/67) of all included papers. Only two interventions have been rolled out nationally – community link workers and welfare advice and health partnerships – with both facing uncertain long-term funding.

**Conclusion:**

There remains a major implementation gap between Scottish Government’s policy ambitions to address health inequalities and sustainable delivery on the ground. To address the ICL, greater overall investment in general practice is needed, together with additional resources for more deprived areas according to local population need (a 'proportionate universalism' approach).

## Introduction

The inverse care law (ICL), coined by Julian Tudor Hart in 1971, states that ‘The availability of good medical care tends to vary inversely with the need for it in the population served’.^1^ The ICL operates in most countries of the world, to varying magnitudes, and more so where there is insurance or payment-based healthcare provision rather than a national health service providing care paid from general taxation and free for all at the point of care.^2^ However, the mismatch of need and supply exists also in the NHS and was demonstrated in Scotland almost 20 years ago, with less funding per patient in practices in deprived areas, despite higher levels of complex multimorbidity and premature mortality.^3^ GPs in deprived areas reported more stress, and patients had poorer outcomes compared with more affluent areas.^4–6^ Since that work, it is unclear what progress there has been in addressing the ICL in Scotland. Although several interventions have been implemented in the past 20 years, there has been no systematic evaluation of what has been tried and what difference it has made.

In 2022 the Health Foundation published an analysis of policies that aimed to improve general practice in deprived areas in England, concluding that policy efforts have been insufficient to overcome the ICL.^7^ Since devolution in 1999, addressing health inequalities has been a stated priority of the Scottish Government,^8^ with several policies to improve general practice in areas of deprivation (See Supplementary Table S1). As part of a wider project on the ICL in Scotland funded by the Health Foundation,^9^ the current authors undertook a systematic scoping review of policies and interventions to address the ICL in Scotland since the year 2000.1

Supplementary Information

**How this fits in**

Recent evidence demonstrates persistence of the inverse care law (ICL) in English general practice, with high health needs but fewer GPs in the most deprived areas; however, there was a lack of similar recent evidence from Scotland. This systematic scoping review found that, since Scottish devolution in 1999, there have been a range of policies and interventions attempting to address health inequalities, with varying success. Despite political ambition to address health inequalities, there is an implementation gap between policy and sustained action; only two of the 20 interventions have been rolled out nationally. To address the ICL, greater overall investment in general practice is needed, together with additional resources for more deprived areas according to local population need (a 'proportionate universalism' approach).

This paper presents the scoping review in greater detail; it focuses on interventions and includes an updated search (from March 2022 to February 2024). The aim was to identify national and local interventions that addressed the ICL in Scottish general practice since 2000, and to review evidence of the impact and sustainability. Initial searches by the research team identified few randomised trials therefore a scoping review methodology was used to allow for the inclusion of different study methodologies and grey literature.^10^

## Method

A systematic scoping review^10^ was undertaken in accordance with the PRISMA extension for scoping reviews.^11^ The initial search was conducted in March 2022 and updated in February 2024. This review is registered with OSF registries (https://doi.org/10.17605/OSF.IO/89WUX).

### Inclusion criteria

Interventions, defined as any activity that aimed to improve mainstream Scottish general practice in areas of socioeconomic deprivation, from 2000 to February 2024 were included in this review. Other primary care services were not included in this review and it did not include interventions focusing on specific inclusion health groups, for example rural populations, sex workers, or migrant populations.

### Search strategy

Searches were conducted on PubMed, Embase, CINAHL, Web of Science, BASE and Cochrane. Headings used were 'general practice', 'Scotland', 'policy'/'intervention', 'quality improvement'/'funding' *and* 'socio-economic deprivation'. The final search syntax is included in the Supplementary Appendix S1. A search of grey literature was also conducted, following the systematic approach of Godin *et al.* ^12^ Supplementary Appendix S1 and Supplementary Table S2 lists the search strategies, and the search engines and websites reviewed.

### Screening and extraction

Papers were uploaded to Covidence 2.0 software. Two reviewers independently screened both title/abstract, and full text. Data was extracted by a single reviewer using a standardised template, with a screen of 10% of papers by the project lead to assess for consistency. Separate templates were used for the published literature and the grey literature. Any disagreements were discussed among the research team and final decisions were made by the project lead.

### Intervention analysis

Included papers were organised by intervention and analysed using the Scottish School of Primary Care’s (SSPC) Evaluation Framework.^13^ This was a two-step process; first, extracting data from included papers to answer a series of questions relating to programme theory and the expected impact at the start of the intervention; and second, investigating the actual impact achieved, the learning from the programme, and whether the intervention achieved spread and sustainability. This produced a narrative account of each intervention within the framework. The research team identified common themes across interventions and summarised these within groups, organised by mechanism. An example of the framework is included in the Supplementary Appendix S1.

### Quality assessment

Quality appraisal of peer-reviewed studies was performed using guidelines from the Joanna Briggs Institute. Papers were scored as the percentage of checklist items that were addressed, and scores were used to assess quality, low if <60%, medium if 60–80%, and high if >80%. Grey literature was not appraised, and no papers were excluded on the basis of quality, as the purpose of the review was to achieve breadth of understanding of all activities undertaken.

## Results

**Figure 1.**
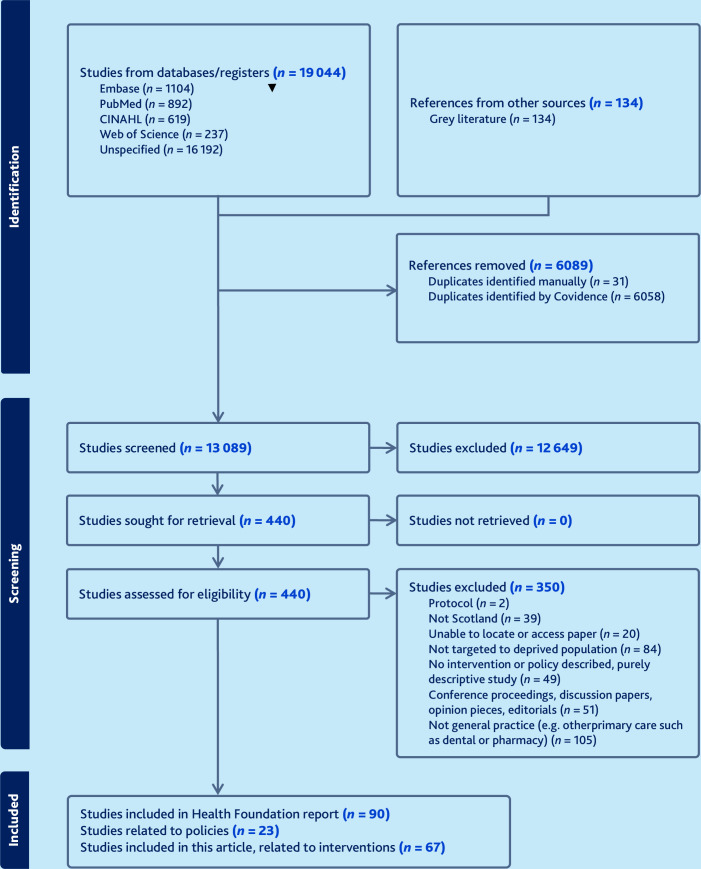
PRISMA diagram for systematic scoping review.

From 13 089 titles, a total of 90 papers were selected for inclusion in the wider project, 44 from peer-reviewed literature and 46 from grey literature (See Figure 1 for PRISMA flow diagram). Of these 90 papers, 67 related to 20 different interventions , and the remaining 23 papers related to policy or strategy documents that are discussed elsewhere.^9^ A summary of all included papers is available as Summary Table S3. For the remainder of this article, we focus on the 67 intervention-related papers (see papers 10-76 in Supplementary Table S3).

A summary of all included papers is available as Supplementary Table S3. For the remainder of this article, the focus is on the 67 intervention-related papers. The broad inclusion criteria were reflected in the range of study designs of included papers, from cluster randomised controlled trials to case studies, with the majority, 55% (*n* = 37), being qualitative or mixed/multimethod designs. The 44 peer-reviewed papers were appraised for quality. 57% (*n* = 25) of the studies were graded as high quality, 36% (*n* = 16) medium quality and the remaining 7% (*n* = 3) articles were of low quality.

Interventions were grouped into four broad categories:

- enhancing patients’ financial or social support;
- targeting specific health conditions;
- holistic interventions that target specific populations; and
- enhancing generalist care.

Table 1 provides a summary of the 20 included interventions, grouped by category, and Supplementary Table S4 provides a fuller overview with further details on programme theory, impact, and sustainability.

**Table 1.** Overview of interventions, categorised by main mechanism of action

Intervention, (papers, *n*), brief description	Impact	Sustainability
**Interventions that enhance financial or social support**		
Community link workers (*n* = 19), practice-attached community practitioners offering non-clinical support, signposting, and health promotion	No difference in patient outcomes with the comparator group. Subgroup analysis demonstrated improved wellbeing for patients who saw the link worker on ≥3 occasions. Correlation between link worker consultation rates and uptake of suggested resources. GPs, link workers, and community organisations reported benefits	Sustained and rolled out nationally. Supported by GPs and patients. Staff are available to fill the role
Embedded welfare advice (*n* = 6), welfare advice workers attached to GP practices to support patients with financial issues such as debt, benefits, and rent arrears	Increased financial gain among eligible patients, demonstrated across disability, child/maternity benefits, as well as debt management and task-shifting away from GPs	Sustained nationally, provides return on investment and staff are available
Green health partnerships (*n* = 1), social prescribing initiatives to promote and support the use of green spaces for health improvement	Green health partnerships have been successfully created but no demonstrated benefit as yet	Currently delivered by third sector and volunteers. Not yet integrated into high-level national strategic plans
Other social prescribing initiatives (*n* = 2), using non-clinical prescriptions for health promotion activities such as exercise classes or social cafes	A complex landscape of roles and responsibilities has emerged across Scotland, with varying levels of evaluation	Funding for social prescribing is complex. Third-sector organisations are also vulnerable to closure, restricting options for referral
**Interventions that target specific health conditions**		
Keep Well (*n* = 7), a national programme to improve cardiovascular anticipatory care in underserved populations	No improvement to mortality demonstrated. Various subjective benefits to staff and patients. Use of an outreach worker was believed to improve patient support and engagement	Owing to the high degree of uncertainty about the evidence supporting health checks, the intervention was not sustained
Blood-borne virus screening (*n* = 3), offering screening for blood-borne viruses to higher-risk groups of patients in general practice	Active screening — either new patients or during routine appointments — increases testing and identifies patients with hepatitis C	Provided funding was in place, screening of new or high-risk individuals could be sustainable
Attached alcohol nurses (*n* = 2), embedding specialist nurses into GP practices to perform targeted assessments and outreach of individuals with problem alcohol use	The pilot was successful in achieving a working partnership between GPs and this ‘specialist primary care service’. The flexible outreach approach was deemed beneficial	If funding for the roles is provided there is no reason to suggest it would not be sustainable. Trained clinical staff are available
**Holistic interventions that target specific populations**		
Starting Well (*n* = 5), combined an intensive health visitor schedule for families living in deprived areas with community development initiatives	Subjective improvements in parent confidence, knowledge, and skills. Complexities identified in the community health component of the health visitor role	Health visitors reported uncovering a large burden of need and escalating workload. The programme was not sustained
Bridge Project (*n* = 1), developed links between older patients in deprived areas and community-based resources to promote health and wellbeing	Respondents were supportive of the intervention and voiced needs for support. Practices organised successful coffee mornings, taster walks and referrals to community organisations	Participants felt that the connections should be sustainable, at relatively low cost. Concerns about dwindling enthusiasm and lack of time — every aspect of the project took longer than expected
Living Better (*n* = 1), qualitative study of mental health issues in patients with long-term conditions, combined with training and resource development	Mental health training for people with long-term conditions and for staff was successfully implemented, with local resources produced	The projects were difficult to get up and running, and there were competing demands for time and resources
Homelessness outreach services (*n* = 1), outreach general practice in homelessness centres to improve accessibility	Subjective improvements to accessing of health care was reported in focus groups	No reference to sustainability, however, the outreach centres did rely on volunteers as well as medical staff
**Interventions that enhance generalist care**		
CARE Plus (*n* = 4), a complex intervention to improve consultations for patients with multiple long-term conditions in areas of deprivation	Increased time for patients in a deprived area was associated with improvements in quality of life and wellbeing. Cost-effective. Patients and staff valued the intervention. Subjective improvements in wellbeing reported	Staff shortages and high workload could affect practice engagement over a longer period
Govan SHIP (*n* = 3), integrated general practice with social work, through MDT meetings, additional GP capacity and colocation of social work	Improved MDT working and GP additional capacity was used in a range of activities, with case reports of benefit	Not sustained owing to concerns about costs. Observed reductions in patient demand suggest the model may be sustainable
Scottish Deep End Project (*n* = 2), a collaboration between academic and frontline GPs working in practices serving the 100 most deprived areas in Scotland	Improved knowledge sharing and support, and advocated for improved services in deprived areas. Implemented the Pioneer scheme. GPs reported less stress and supported the programme	Ongoing since its inception in 2009, supported by GPs, RCGP, and Scottish Government
Deep End Pioneer Scheme (*n* = 2), a fellowship scheme for early career GP Fellows in Deep End practices, also provided protected time for experienced GPs to pursue service development and collaborative working	Qualitative improvements to confidence and knowledge. Supported by participants	Only two cohorts of Fellows completed the scheme, which ended as the COVID-19 pandemic struck. There is support for relaunching this scheme
New models of primary care (*n* = 1), a range of primary care test-of-change projects, some of which aimed to address health inequalities	Qualitative improvement to patient empowerment and resource utilisation for social prescribing	Sustainability and spread relied heavily on both time and cost-saving strategies and ongoing support from practitioners
Local healthcare cooperatives (*n* = 2), voluntary groups of GP practices within geographical areas to manage budgets, undertake commissioning, and pursue quality improvement	Practices in deprived areas or practices with difficulty accessing local authority care homes were more likely to join a local healthcare cooperative. It is not clear if joining a local healthcare cooperative had a demonstrable benefit	Relies on political support and funding. They have since ceased to operate and been replaced by health and social care partnerships
Training for healthcare staff (*n* = 2), a range of health inequalities-specific training available to healthcare staff, for example, Health Inequality Fellowships	Community development training course did not have a major impact on the work of participants. Other training not assessed formally	No specific reference to sustainability, any training programmes would need to be repeated regularly
Participatory action research (*n* = 1), involving a deprived population in shaping quality improvement of local primary care services	Participatory action in one community was possible, however, there was modest impact on service delivery	It is likely that such action would need to be regularly repeated in specific localities and therefore sustainability may be hard to achieve
Infrastructure (*n* = 2), examples of infrastructure improvements (for example premises) that were made in areas of deprivation	Proposed infrastructure only	Long-term sustainability would be achieved with appropriate investment

MDT = multidisciplinary team.

### Interventions than enhance financial or social support

There were 28 papers relating to four interventions classified as enhancing financial or social support. They were the community link worker (CLW) programme (*n* = 19), embedded financial advice (*n* = 6), green health partnerships (*n* = 1), and other social prescribing initiatives (*n* =2). These interventions created new non-clinical roles with the purpose of improving health behaviours or supporting patients with non-clinical interventions.

The CLW programme is one of the main interventions in Scottish general practice that aims to address health inequalities. Starting as a pilot project in seven Deep End practices in Glasgow in 2014, link workers have been rolled out across Scotland, with more than 300 CLWs now working in general practice, mostly in areas of socioeconomic disadvantage. Included studies demonstrate broad support for the CLW programme among clinicians, patients, and community organisations with some evidence of benefit to patients.^14–19^

Welfare advice and health partnerships involve embedding welfare advisers into general practices, which encourages openness about financial issues.^20,21^ One evaluation reported a return of £25 for every £1 invested.^22^ The benefits of green health partnerships and other social prescribing initiatives for patients in deprived areas is less clear, and initiatives were often located in affluent areas.^23–25^

In terms of sustainability, several factors were identified that suggested both social prescribing initiatives and embedded welfare advisers could be sustainable. They are acceptable to patients and GPs,^15,17,26^ have potential to reduce workload in general practice,^16,26^ skilled practitioners are available, and they are typically employed from within communities.^27^ However, funding is precarious,^28^ and third-sector resources are also vulnerable to the challenging economic climate.

### Interventions that target specific health conditions

There were 12 papers related to three interventions classified as vertical or screening programmes targeting specific health conditions. They were Keep Well (*n* = 7), blood-borne virus (BBV) screening (*n* = 3), and attached alcohol nurse specialists (*n* = 2). These interventions used case-finding, targeted screening, and outreach to improve specific health conditions.

Launched in 2006, Keep Well was a national programme of anticipatory care that aimed to reduce cardiovascular morbidity and mortality by providing health checks to people aged 45–64 in primary care settings in deprived areas. It failed to demonstrate an impact on cardiovascular disease,^29,30^ but made effective use of outreach staff to convert ‘non-attenders’ to ‘engagers’.^31–33^ There was considerable variation in how Keep Well was implemented. Central funding was stopped in 2017.

The papers describing BBV screening,^34–36^ and practice-attached alcohol nurses,^37,38^ found that they were effective at identifying and engaging the target populations. The involvement of alcohol nurses led to increased engagement with alcohol treatment, reduced GP contact, and reduced hospitals admissions. Feedback from people who used the alcohol nurses service and staff was also positive. However, Keep Well and the attached alcohol nurses was not sustained and there was no evidence that the BBV screening was sustained or led to a change in practice.

### Holistic interventions that target specific populations

There were eight papers related to four holistic interventions that targeted specific, underserved populations: Starting Well (*n* = 5), the Bridge Project (*n* = 1), Living Better (*n* = 1) and homelessness outreach services (*n* = 1). The interventions aimed to improve access to health care or community resources for underserved individuals and families.

Starting Well (2001–2005) was a child health demonstration project for families living in disadvantaged areas, combining an intensive health visitor schedule with community development initiatives. It led to increased parental confidence and reduced anxiety,^39^ but variable levels of enthusiasm and engagement by primary care teams affected implementation.^40,41^

The Bridge Project and Living Better were pilot interventions that targeted older people^42^ and people living with long-term conditions,^43^ respectively, and the homelessness outreach service successfully engaged with people experiencing homelessness.^44^

Workload and time-pressures were challenging even in these well-funded pilots, as staff uncovered a large burden of unmet need – another facet of the ICL.^45^ In Starting Well, the health visitors found ‘community development’ complex and hard to achieve and felt that a dedicated position should be created to enable this, rather than integrating it into existing staff roles.^45^

### Interventions that enhance generalist health care

There were 19 papers related to nine interventions which enhance generalist health care: CARE Plus (*n* = 4); Govan SHIP (*n* = 3); the Scottish Deep End Project (*n* = 2); Deep End Pioneer Scheme (*n* = 2); new models of primary care (*n* = 1); local healthundercare cooperatives (*n* = 2); training for healthcare staff (*n* = 2); participatory action research (*n* = 1); and infrastructure developments (*n* = 2). These interventions were more varied in their programme theories, but there was commonality around patient-centred care and enablement, increased GP time and capacity, improved multidisciplinary team (MDT) working, and supporting GPs working in deprived areas.

Several of these interventions have been effective at increasing the supply or quality of general practice in deprived areas, directly addressing the ICL. The Deep End Pioneer Scheme recruited early career GPs to areas of deprivation,^46,47^ the CARE Plus study (involving continuity of care and longer consultations for people with multiple long-term conditions) was found to be cost-effective^48^ and Govan SHIP demonstrated the value of extended MDT working between health and social care staff.^49,50^ All these involved additional time and capacity for GPs in deprived areas, enabling beneficial activities such as complex case reviews, and sharing of learning within and between practices. Ongoing funding was the main barrier to sustainability, with qualitative work revealing the negative impact this had on staff morale and buy-in.^13,47^

## Discussion

### Summary

This review identified 67 papers related to 20 different interventions that have been implemented in Scottish general practice in the past 20 years, with the aim of addressing health inequalities. Some directly addressed the ICL by increasing the volume or quality of general practice in deprived areas, whereas others referred to health inequalities but lacked detail on mechanisms of action. Common themes among effective interventions were: increasing clinician time or capacity to engage with complex clinical work; embedding new roles within general practice such as CLWs or welfare advisers; use of outreach and system flexibility to better engage with underserved individuals; creating specific roles for coordinating community development, bridging between health care and third sector; and effective MDT working and staff buy-in. Across all interventions, increasing workforce capacity, introducing clinical and non-clinical roles and improved funding were able to directly address the ICL, making the case for proportionate universalism whereby services are funded proportionate to need by deprivation.

Funding was the main barrier to sustainability, and it was not always clear why funding was stopped. Typically, Government expected that health boards would take over the funding for successful projects, but this was not always possible because of financial constraints. Larger national interventions such as Keep Well were not able to demonstrate sufficient benefit to warrant ongoing investment.^30^ Relatively small-scale interventions or pilot projects were limited by short timescales making it difficult to demonstrate impact or generalisability.

Interventions that enhanced financial or social support had the strongest evidence to support them.^19,51,52^ However, there were also interventions that did not have quantitative evidence of benefit but did have strong qualitative support and provided accounts of positive impact for individual cases.^30,38,44,45,49^ Others demonstrated a roadmap for implementation, akin to a feasibility study.^36,43,47,49^ For example, Govan SHIP demonstrated improved integration with social work to the benefit of staff and patients.^49^ The impact of the Scottish Deep End Project is difficult to measure, but it has been a driving force behind many of the interventions, including CLWs and welfare advisers, both of which started as Deep End pilots.^9,20,53^

### Strengths and limitations

A broad search strategy across databases and grey literature sources was undertaken. The inclusion of non-peer-reviewed literature limited the ability to formally assess quality across all included papers. There were few high-level study designs; there were two randomised controlled trials,^19^ and the majority were uncontrolled before and after studies. The quality of the grey results was variable – however, most employed qualitative or mixed-methods.

A robust methodology was followed for grey literature reviews, which included consultation with experts to identify missed work.^12^ The search may have been improved using snowballing or searching reference lists to identify any further papers that were missed. However, it is unlikely this would have yielded additional insights.

A common weakness of the interventions was that where they were rolled out across different sites, there was variation in implementation, limiting generalisability. This work did not consider intervention type as this is not part of the Scottish School of Primary Care evaluation framework. Future work could assess interventions using a framework such as the Eric taxonomy, which could reveal what intervention types have worked, and which have not been trialled.^54^

This review focused on general practice; future research should evaluate how the ICL manifests more broadly in primary care, including community nursing, dentistry, pharmacy, and allied health professionals.

### Comparison with existing literature

In 2022, the Health Foundation published an analysis of policies in England that were designed to improve general practice in deprived areas.^7^ They found that efforts to reduce inequities in the provision of GP services over the past 30 years have been insufficient. The current work followed a similar process, but took a broader view of the ICL, encompassing not only the supply of GPs and funding in deprived areas — the focus of the English report — but also including interventions that have sought to improve the quality of care in these practices.

In 2023, Gkiouleka *et al* published a realist review of interventions to address inequalities in general practice. They took a broad definition of health inequalities to include patient, system, and health outcomes, and focused on underlying programme theories. They also found that increasing funding in deprived areas can increase staffing and increase clinical capacity, and concluded that intersectional, flexible, and connected interventions promote equitable practice.^55^

A 2021 Lancet series on the 50th anniversary of Tudor Hart’s seminal paper summarised evidence of the ICL in the UK and internationally.^56^ Globally, investing in universal health care and strengthening primary care, proportionate to need, can improve health equity.^2^ In the UK, the 1970s allocation formula reduced geographical inequality in hospital expenditure,^57^ and the 2000s saw strengthened primary care provision.^56^ Lessons can also be drawn from efforts to increase the workforce in underserved and rural areas, for example, by recruiting from these areas, including rural placements in training and utilising financial incentives.^58^

The Deep End movement has now spread from Scotland to a total of 20 sites across the UK and internationally.^59^ This is leading to a growing evidence base of ways to address the ICL in primary care. In Canada, the SPARK tool has been used to collect social determinants data,^60^ in Northern Ireland work is being done to improve training and education in areas of deprivation,^61^ and in Ireland further work has investigated the effectiveness of link workers.^62^

### Implications for research and practice

This work identifies a range of interventions which have sought to improve general practice in areas of deprivation, two of which — CLW and welfare advisers — have been rolled out across Scotland. Evidence of impact is limited, so future research should prioritise robust evaluation of these interventions. In March 2023, the Scottish Government developed the Inclusion Health Action in General Practice programme, funding GPs to improve community connections, enhance workforce knowledge and skills, and provide outreach and extended consultations.^63^ This current reviews findings support this approach, and the authors of this review endorse its ongoing funding and robust evaluation.

Drawing on lessons from other countries, there is clear scope in the UK for improving systems for collecting and analysing detailed sociodemographic data to identify health inequities; adopting a health equity approach to all work, including advocacy; and developing and testing tools and interventions to improve the response to socioeconomic disadvantage, with rigorous evaluation to assess implementation and effect.^64^

In conclusion, there remains a major implementation gap between Scotland’s policy ambitions and sustainable delivery on the ground. To address the ICL, policymakers should support greater overall investment in general practice, together with additional resources for more deprived areas according to local population need (a ‘proportionate universalism’ approach). By proactively engaging with community resources, adopting flexible systems, and embracing extended, patient-centred consultations, GPs can make a tangible difference for those experiencing socioeconomic disadvantage.

### Registration

This review is registered with OSF registries, https://doi.org/10.17605/OSF.IO/89WUX
