# Supplementary material for: Addressing the inverse care law in Scottish general practice: systematic scoping review
Source: Br J Gen Pract. 2025 Jul 14;75(757):e549–58. doi: 10.3399/BJGP.2024.0622 (PMC12729052; doi:10.3399/BJGP.2024.0622)
Supplement: Supplementary file 1 — Supplementary Information [file bjgp-75-757-0622-suppl.pdf]

Supplementary Table S1: Policies relevant to addressing health inequalities in Scottish general practice

Year	***Title* and summary**
2005	*Delivering for Health* [1]<br>Outlined actions for NHS Scotland to achieve health improvement, including plans for a programme of anticipatory care (Keep Well). Emphasised the integration of GP with other community services. Stated "*We believe the most significant thing we can do to tackle health inequalities is to target and enhance primary care services in deprived areas*".
2011	*The Christie Commission* and *Scottish Government response* [2]<br>Review of public services in the context of tightening budgets. Called for public service reform, not specific to general practice but highly influential for future health and social care policy.
2016	*A National Clinical Strategy for Scotland* [3]<br>Framework for the development of health services across Scotland for the next 15 years, including stronger primary care, Multi-Disciplinary Team (MDT) working and more integration. Emphasised the need for Realistic Medicine [4].
2017	*Improving Together: A National Framework for Quality and GP Clusters in Scotland* [5]<br>Framework for values-driven quality improvement via GP clusters. Outlines the intrinsic and extrinsic roles of clusters, including "*improving wellbeing, health and reducing health inequalities*".
2017–2018	*National Health and Social Care Workforce Plan: Parts 1 & 3* [6,7]<br>Outlined a strategy for recruiting to (and from) areas of deprivation, including plans to employ 250 Community Link Workers in deprived areas, along with 800 more GPs nationally over the subsequent 10 years. Requirement for primary care improvement plans to show how inequalities will be addressed.
2019	*National Monitoring and Evaluation Strategy for Primary Care in Scotland* [8]<br>Detailed the Primary Care Outcomes Framework as a conception and evaluation tool intended to be used to understand changes in the subsequent ten years.
2022	*Report of the Primary Care Health Inequalities Short-Life Working Group* [9]<br>Made 23 recommendations for actions in primary care to reduce health inequalities, including support for Community Link Workers and welfare advisers in practices, an enhanced service to target additional resources to general practices in areas of the highest deprivation and need, and health-equity focused training for health and social care staff.

Supplementary Table S2: List of the websites reviewed following the grey literature search methods.

Grey Databases	Government	Universities	NHS	Third sector/Other
ETHOS<br>BASE<br>Cochrane<br>Greynet<br>Zetoc<br>JISC library hub<br>Social Care Online<br>Social Science research network	Department of Health and Social Care<br>Health Improvement Scotland<br>Scottish Public Health Network<br>Glasgow Centre for Population Health<br>National Institute of Health Research<br>National Institute for Health and Care Excellence<br>Health Protection Scotland	University of Glasgow<br>University of Edinburgh<br>University of Stirling<br>University of Dundee<br>University of Aberdeen<br>University of St Andrews<br>University of Strathclyde	Public Health Scotland<br>NHS Health Board websites (14 in total)<br>NHS Education for Scotland<br>NHS Health Scotland	Kings fund<br>Nuffield Health<br>Health Foundation<br>Health and social care Alliance<br>Voluntary Health Scotland<br>RCGP Scotland<br>Queens Nursing Institute Scotland

Supplementary Table S3: Summary of Included studies, reports and policies.

Title	Author/Organisation (Year)	Source	Aim	Study Design	Settings/Participants	Main Outcome(s)	JBI Scores
Link worker Programme							
Implementing social prescribing in primary care in areas of high socioeconomic deprivation: process evaluation of the 'Deep End' community Links Worker Programme.	Chng et al. (2021) [10]	Database search	To explore the implementation process of the link worker programme (LWP).	Qualitative study.<br><br>Qualitative process evaluation of the 'Deep End' LWP over a 2-year period.	7 General Practices in deprived area of Glasgow.	Only three practices fully implemented the LWP. Fully integrated practices had better shared understanding of the programme among staff, higher staff engagement with the LWP, and were implementing all aspects of the LWP at patient, practice, and community levels of intervention.	8/10<br><br>80%
Does Self-Determination-Theory help explain the impact of social prescribing? A qualitative analysis of patients' experiences of the Glasgow 'Deep-End'	Hanlon et al. (2021) [11]	Database Search	To explore the utility of self-determination theory in understanding the reported benefits of the LWP.	Qualitative study. Thematic analysis of semi-structured interviews with 12 patients referred to Community Links Practitioners using Self-Determination	12 patients referred to community links practitioners.	Four patients described no overall change in daily life, two described slight improvement and six described moderate or major improvement.	8/10<br><br>80%

Community Links Worker Intervention.				Theory as a framework.			
"The state They're in": Unpicking fantasy paradigms of health improvement interventions as tools for addressing health inequalities.	Mackenzie et al. (2020) [12]	Database search	To explore the 'fantasy paradigm' of addressing inequality at an individual level and not the socio-political level, and to identify opportunities for deepening practitioner understandings of the socio-political determination of health.	Qualitative Study. In-depth interviews with 47 professionals involved in delivering the LWP.	Seven GP practices had been randomly allocated to the LWP and eight formed the comparison group.	Narratives highlighted different explanations of how intervention could tackle health inequalities including: firm commitment to individualised approaches; hopeful pessimism; the social-determinants-of-health as an un-politicised and nondeterministic backdrop to poor health; and finally, incomplete understanding of the social gradient as a population concept.	7/10<br><br>70%
Effectiveness of Community-Links Practitioners in Areas of High Socioeconomic Deprivation.	Mercer et al. (2019) [13]	Database search	To assess the effect of a primary care–based community-links practitioner (CLP) intervention on patients' quality of life and well-being.	Cluster-randomised trial.<br>Data were collected on 288 and 214 (74.3%) patients in the intervention practices at baseline and follow-up, respectively, and	15 deep end practices in Glasgow	Intention-to-treat analysis found no differences between the 2 groups for any outcome. In subgroup analyses, patients who saw the CLP on 3 or more occasions (45% of those referred) had significant improvements in EQ-5D-5L, HADS-D, HADS-A, and exercise levels.	8/9<br><br>89%

				on 612 and 561 (92%) patients in the comparison practices.			
Delivering a primary care-based social prescribing initiative: a qualitative study of the benefits and challenges.	Skivington et al. (2018) [14]	Database search	To investigate issues relevant to implementing the LWP to improve inter-sectoral working to achieve public health goals.	Qualitative study. Qualitative interview study with community organisation representatives and CLPs in LWP areas.	Six CLPs and 30 Community Organisation Representatives	Participants identified some benefits of collaborative working, particularly the CLPs' ability to act as a case manager for patients, and their position in GP practices, which operated as a bridge between organisations.	8/10<br><br>80%
Community Links: Perspectives of community organisations on the Links Worker Programme pilot and on collaborative working with primary health care.	Smith & Skivington. (2015) [15]	Google search engine	To explore the perspectives of community organisations who receive referrals from CLPs.	Qualitative study. Qualitative interview study with community organisation representatives and CLPs in LWP areas.	Six CLPs and 30 Community Organisation Representatives	Advantages were identified, such as the CLP role as a case manager, improved referrals and bridging between organisations and GP practices. The main issues were capacity/funding, and organisational processes and attitudes.	Not peer reviewed
Links Project Report-Developing the connections between General Practices and their communities.	Scottish Government. (2012) [16]	Targeted website search	To develop and test a sustainable local model to improve links between General Practice and community support.	Non-randomised experimental study with service improvement methodology.	6 practices in Glasgow and 4 practice in Fife, in deprived areas.	35 out of 131 patients were using a service recommended by a GP. Improved relationships between community organisations and	Not peer reviewed

						General Practice could have a positive impact.	
Evaluation of New Models of Primary Care in Scotland: Ayrshire and Arran Case Study	O'Donnell et. al (2019) [17]	Targeted website search	To implement tests of change in Primary Care. One example is employing CLPs	Test of change	No specific details are given.	States that link workers are operational but gives no further details and evaluation plans are to be decided.	Not peer reviewed
Link Worker Programme Record of Learning Series 2: Social Determinants in Primary Care	Alliance. (2016) [18]	Targeted website search	To detail the most common issues that CLPs are supporting people to address.	Multi methods case study, including summary of referral data and informal discussion with CLPs.	7 Deep End GP practices	The role of CLP enhances capacity for addressing social issues. This is achieved both through building capacity among the wider practice teams for appropriate signposting to take place and the availability of the CLP to undertake one-to-one work with individuals who require more support.	Not peer reviewed
Developing a Culture of Health: The role of signposting and social prescribing in improving health and wellbeing	Alliance. (2017) [19]	Targeted website search	A report on social prescribing in Scotland.	Descriptive cross-sectional report.	Various social prescribing initiatives are described. The LWP is relevant to the ICL.	Summarises initiatives across Scotland, pulling together evidence and qualitative insights.	Not peer reviewed
Links Worker Programme, Record of Learning, Module:	Gourley & Charlton. (2015) [20]	Targeted website search	To detail the development and	Narrative account of the processes, decisions and	7 Deep End practices in Glasgow.	The LWP is created and recruitment of CLPs commences.	Not peer reviewed

Context and Creation of the Programme			implementation of the LWP.	actions involved in developing and implementing the programme.			
Monitoring and evaluation of primary care in Scotland: the baseline position	Wyper et al. (2020) [21]	Targeted website search	To provide a baseline position and relevant trend information to support monitoring and evaluation of primary care reform. One reform described is the LWP	Cross-sectional data in Scotland and creation of an outcomes framework.	Commitment to deliver 250 CLPs.	Produces an outcomes framework for monitoring and evaluation.	Not peer reviewed
Learning from the community link worker early adopters.	Public Health Scotland. (2020) [22]	Targeted website search	To understand the implementation and delivery of the LWP in early adopter sites, to inform on-going delivery.	Qualitative fieldwork of 11 interviews and 7 focus groups.	5 early adopter sites	Among a number of themes, they highlight the need for clarity of roles, the enablers of multi-disciplinary team working, and some of the practical challenges relating to available space, IT, and monitoring and evaluation.	Not peer reviewed
VHS Gold star exemplars. Third sector approaches to Community Link Working across Scotland.	VHS & SJ Services (2017) [23]	Targeted website search	To identify the number and range of LWPs in Scotland and to inform he Government's development of a national programme.	Multi methods. Employs scoping interviews, online surveys and 3 case studies.	Scotland-wide	Summarises the range of programmes, operational issues and outcomes. Makes recommendations to Scottish Government.	Not peer reviewed

Improving links in primary care: project report	Alliance & RCGP. (2014)[24]	Targeted website search	to test the feasibility of embedding 'A Local Information System for Scotland' (ALISS) for possible future roll out across Scotland.	Mixed-methods.	4 General practices in Scotland	Identified 3 methods of improving links in primary care: mapping assets, using ALISS and adopting a link worker approach.	Not peer reviewed
Glasgow 'Deep End' Links Worker Programme Practice Audit Questionnaire Evaluation	Alliance (2016) [25]	Google search	To evaluate the perceived effectiveness of the LWP	Audit Questionnaire.	125 responded out of 551 patients referred to a CLP	2/3rds of patients reported that they found contact helpful, and 60% reported some improvement in well-being.	Not peer reviewed
Essential Connections: Exploring the range and scope of community link worker programmes across Scotland	Smith (2023) [26]	Google search (update)	To document and learn from the current range of CLW programmes across Scotland.	Mixed methods (survey and interviews).	61 CLWs for the survey and 18 CLW programme leads for the interviews	The strength of CLWs in helping to tackle the impact of health inequalities at an individual level was widely recognised. There was less certainty regarding the contribution CLW programmes made towards tackling wider systemic health inequalities.	Not peer reviewed
Community Link Worker Programme-Mental Health and Wellbeing Deep Dive	Mekina & Conduit-Turner (2022) [27]	Targeted website search (update)	To better understand the scale at which mental health featured in appointments in a given time period, the range of issues that	Cross-sectional report.	70 CLW	The evidence presented from this exploratory work indicates that there is an undoubted need for Community Link Workers' provision of support in relation to patients' mental health and wellbeing, with the	Not peer reviewed

			CLWs respond to in relation to mental health and wellbeing and associated issues and/or challenges for CLWs in relation to supporting this work.			vast majority of patients that were seen primarily and explicitly requiring mental health support.	
NHS North Highland Community Link Worker Project- Year 1 Report	NHS Highland (2023) [28]	Targeted website search (update)	To look at the community link worker service delivery from 1st May 2022 to 30th April	Surveys	131 clients who started with a CLW	Most clients had a significant positive change in their mental health and well-being. However, there are still practical challenges for the CLW such as recruitment, accommodation and wifi access.	Not peer reviewed
Embedded Welfare Advice							
Nurse-led welfare benefits screening in a General Practice located in a deprived area	Hoskins and Smith (2002) [29]	Database search	To evaluate a nurse-led attendance allowance screening service.	Non-randomised experimental study	One General Practice located in two sites in deprived areas of Glasgow	Thirty-seven of the original 86 participants plus four relatives were found not to be claiming the benefit payments that they were entitled to. There were unclaimed benefits to a total of £112,893.	3/9<br><br>34%
Roll-out of a nurse-led welfare benefits screening service	Hoskins et al. (2005) [30]	Database search	To evaluate the roll-out of a nurse-led Attendance	Non-randomised experimental study	24 General Practices	Three hundred and sixty-three participants and 13 relatives	3/9<br><br>34%

throughout the largest Local Health Care Co-operative in Glasgow: An evaluation study			Allowance (AA) screening programme			were awarded a total of £1,136,424.10.	
Evaluation of New Models of Primary Care in Scotland: Tayside Case Study	Sullivan et al. (2019) [31]	Targeted website search	To determine the outcomes of primary care tests of change. One example is embedded welfare advice	Test of change employing mixed-methods.	8 GP practices across Dundee	Improved the efficiency of benefits claims resulting in financial gain of £2,033,500.	Not peer reviewed
The Deep End Advice Worker Project: embedding an advice worker in general practice settings	Sinclair (2017) [32]	Google search	To present the learning from the delivery of an embedded advice service	Quality improvement methodologies using mixed-methods.	2 GP practices in Glasgow	Median financial gain of £6,967.96 across 167 successful claimants.	Not peer reviewed
Integrating money advice workers into primary care settings: an evaluation	Egan & Robinson (2019) [33]	Targeted website search	To explore the money advice case outcomes, and to describe the set up and delivery of the project	Mixed-methods.	3 advice workers across 9 Deep end practices in Glasgow	The project had £1.5 million in gains across 654 people seeking advice. The project returned £25 on every £1 invested.	Not peer reviewed
Building Connections: co-locating advice services in general practices and job centres	Sinclair (2017) [34]	Targeted website search	To evaluate the co-location of financial and employment advice services	Quality improvement methodologies using mixed methods.	Implemented across 2 job centres and 2 GP Practices	There were 707 referrals, £992, 778 worth of financial gain and £212, 831 worth of debt identified, and	Not peer reviewed

						management plans put in place.	
Green Health Partnerships							
Green health partnerships in Scotland; pathways for social prescribing and physical activity referral	McHale et al. (2020) [35]	Database search	To explore the views and experiences of professionals involved in green health partnerships	Qualitative study with focus group and semi-structured interviews.	A total of 5 focus groups and 14 interviews	This study demonstrated success in the creation of partnerships to develop green health interventions that can integrate with social prescribing.	9/10<br><br>90%
Other Social Prescribing Initiatives							
Weathering the storm: A qualitative study of social prescribing in urban and rural Scotland during the COVID-19 pandemic	Fixsen et al. (2021) [36]	Database search	To understand the response of 3 social prescribing schemes to the Covid-19 pandemic	Qualitative study.	23 stakeholders in 3 social prescribing schemes	Findings revealed a complex social prescribing landscape in Scotland with schemes funded, structured and delivering services in diverse ways.	8/10<br><br>80%
Challenges and Approaches to Green Social Prescribing During and in the Aftermath of COVID-19: A Qualitative Study	Fixsen & Barrett. (2022) [37]	Database search (update)	To examine the challenges and opportunities of delivering green social prescribing during and in the aftermath of COVID-19,	Qualitative study.	35 interviews with stakeholders in Scotland and Northeast England	Stakeholders were in general agreement about the benefits of nature-based interventions. Link Workers were more circumspect about suggesting outdoor activities, pointing out both psychological and practical obstacles.	8/10<br><br>80%
Keep Well							

The outreach worker role in an anticipatory care programme: A valuable resource for linking and supporting.	Carver et al. (2012) [38]	Database search	To understand how staff and patients view the Keep Well outreach worker role	Qualitative study (interviews)	12 Keep Well staff and 4 patients	The outreach worker role was viewed positively, it reduced pressure on clinical staff and patients appreciated the support to make lifestyle changes.	8/10<br><br>80%
'It's just a way of approaching things now': Staff perspectives of an anticipatory care programme in Edinburgh	Carver et al. (2012) [39]	Database search	To understand how staff view changes in practice resulting from the implementation of Keep Well.	Qualitative study (interviews)	12 Keep Well staff	Keep Well was viewed positively, especially in terms of increased knowledge and skills for staff and the benefits of a holistic health check to patients. Staff believed that Keep Well has improved their practice beyond the project.	8/10<br><br>80%
Delivering a national programme of anticipatory care in primary care: A qualitative study	O'Donnell et al. (2012) [40]	Database search	To explore the issues and tensions underpinning the implementation of a national programme of anticipatory care.	Qualitative study (semi-structured interviews)	118 interviews were conducted at 5 Keep Well test sites and with key stakeholders	Four underlying tensions were identified. First, those between a patient-focused general practice approach versus a population-level health-improvement approach, linking disparate health and social services; secondly, medical approaches versus wider social approaches; thirdly, a population-wide approach versus individual targeting;	8/10<br><br>80%

						and finally, reactive versus anticipatory care.	
Using outreach to involve the hard-to-reach in a health check: What difference does it make?	Sinclair and Alexander (2012) [41]	Database search	To understand how outreach helped to achieve health screening attendance amongst 'hard-to-reach' groups	Qualitative study (semi-structured interviews)	30 individuals who initially failed to attend for health screening but were subsequently appointed after outreach intervention	Not all non-attenders for screening appointments are negatively disposed towards health screening and defining them all as 'hard-to-reach' does them a disservice. The majority appeared to need outreach staff to convert them into attenders.	8/10<br><br>80%
Evaluation of 'Keep Well' programme in NHS Greater Glasgow & Clyde	Scoular (2012) [42]	Targeted website search	To evaluate Keep Well, an anticipatory care programme with the aim of reducing cardiovascular disease and its risk factors among 45–64 year-olds.	Integrated evaluation framework	Greater Glasgow and Clyde	Keep Well has failed to demonstrate the effectiveness or efficiency of Keep Well as a cardiovascular intervention. However, there are promising process indicators.	Not peer reviewed
Exploration of the Community Health Outreach Worker and Health Case Manager roles	FMR Research (2010) [43]	Targeted website search	To explore the roles of Community Health Outreach Worker (CHOW) and Health Case Manager (HCM) in Keep Well	Mixed-methods (database analysis, semi-structured interviews, focus groups and structured interviews).	Stakeholders, staff, primary care clinicians and patients in Glasgow.	CHOWs had a low level of contact with a large number of patients, HCMs had the reverse. It was not clear if it addressed the ICL, as participants who were engaged required a large amount of time and effort and	Not peer reviewed

						therefore other referrals received little attention.	
The impact of Keep Well: An evaluation of the Keep Well programme from 2006 to 2012	NHS Health Scotland (2014) [44]	Targeted website search	To provide a 'pragmatic evaluation' of Keep Well	Mixed-methods (Qualitative study of implementation and quantitative analysis of trends in cardiovascular disease).	Scotland-wide	Roll-out was adapted locally. Impact on health was inconclusive across sites. Keep Well did not make a difference to deaths and illnesses.	Not peer reviewed
Blood-borne virus screening							
Evaluation of a General Practice Based Hepatitis C Virus Screening Intervention	Anderson et al. (2009) [45]	Database search	To evaluate an intervention which offered screening to individuals who attended non-urgent appointments.	Non-randomised experimental study (intervention versus comparison practice).	Two practices in a deprived area of Glasgow	Of 584 eligible attendees, 421 (72%) were offered and 117 (28%) accepted testing in the intervention practice; no testing was undertaken in the comparison practice.	7/9<br><br>78%
Learning lessons to improve blood borne virus testing in primary care in Scotland	Bell et al. (2018) [46]	Database search	The practice's new patient registration form was modified to include an offer of BBV testing, with patients asked to indicate whether they wished to opt in to having a test.	Non-randomised experimental study.	One practice in the North-East of Scotland	A total of 1399 new patients were eligible for testing and included in the final analysis. 188 individuals were tested during the pilot period, 13.4% of the eligible patients.	6/9<br><br>67%

Identifying former injecting drug users infected with hepatitis C: an evaluation of a general practice-based case-finding intervention	Cullen et al. (2012) [47]	Database search	Hepatitis C screening was offered to attendees with a history of intravenous drug use.	Non-randomised experimental study.	8 intervention practices and 8 control practices in deprived areas of Glasgow	Of 422 eligible intervention practice attendees, 218 (52%) were offered an HCV test and, of these, 121 (56%) accepted. In control practices, 8 (22%) of 36 individuals tested were antibody positive.	8/9<br><br>89%
Attached alcohol nurse specialists							
Deep End Report 31- Attached Alcohol Nurse<br>Deep End Pilot (July 2015–2016): final report	Williamson & Craig (2016) [48]	Targeted website search	To address the needs of people with problem alcohol use who are in contact with their general practices but who have not previously engaged well with addiction services.	Non-experimental study.	Two full-time equivalent specialist band 6 nurses working across 6 deep end practices in Glasgow	132 patients were referred, 71% of those agreed to be seen, and 82 patients had specialist alcohol assessment and treatment in the pilot.	Not peer reviewed
Primary Care Alcohol Nurse Outreach Service (PCANOS) Full Evaluation	King (2022) [49]	Consultation with experts	To address the needs of people with problem alcohol use who are in contact with their general practices but who have not previously engaged well with	Non-experimental study, using multi-methods (referral data, service user and GP feedback and audit forms).	143 practices had access to PCANOS	PCANOS is targeting appropriate patients, has positive outcomes of increased engagement with alcohol treatment, reduced GP contact and reduced ED and hospital admissions. Feedback was positive.	Not peer reviewed

			Alcohol and Drug Recovery Services (ADRS).				
Starting Well							
Benefit or burden: Introducing paraprofessional support staff to health visiting teams: The case of Starting Well	Mackenzie (2006) [50]	Database search	To elicit views in Starting Well relating to the use of paraprofessional staff	Qualitative study (semi-structured interviews)	18 managerial staff and 33 health visitors and health support workers in Glasgow	Health Support Workers were successfully implemented into the Starting Well programme and were widely perceived to be beneficial. There were complexities from dual management, and challenges from deploying potentially vulnerable staff.	7/10<br><br>70%
'Doing' public health and 'making' public health practitioners: Putting policy into practice in 'Starting Well'	Mackenzie (2008) [51]	Database search	To understand the implementation and operationalisation of Starting Well, in reference to developing the public health role of Health Visiting	Qualitative study (semi-structured interviews)	44 staff responsible for developing and implementing the intervention in Glasgow	The policy context for public health demands that increasing numbers of health workers are familiar with its principles and modus operandi. It was contested as a role for health visitors, staff maintained a clinical approach.	8/10<br><br>80%
The process of health visiting and its contribution to parental support in	McIntosh & Shute (2007) [52]	Database search	To understand how the process of health visiting resulted in	Qualitative study (semi-structured interviews)	20 mothers and their health visitors in Glasgow	Parental perceptions of being supported were exemplified by increased confidence in infant care, reduced anxiety	8/10<br><br>80%

the Starting Well demonstration project			parents' perceptions of being supported.			regarding infant care needs, increases in knowledge and sense of personal competence in parenting practices, reduced isolation, and advocacy for those experiencing problems.	
The Starting Well Health Demonstration Project	Ross et al. (2005) [53]	Database search	To demonstrate that child health can be improved by a program of activities that both supports families with intensive home visiting and provides them with access to enhanced community-based resources	Mixed-methods (quantitative and qualitative methods using a theory of change)	1156 families in two areas of Glasgow	Demonstrates successful implementation of the programme. Full evaluation is outlined.<br>They found that health visitors found it hard to maintain the community development aspect of the role with the clinical commitment.	6/6<br><br>100%
Targeting health visitor care: Lessons from Starting Well	Wright et al. (2008) [54]	Database search	To assess how accurately family needs can be predicted by health visitors before 4 months of age	Non-randomised experimental study (population cohort study)	1202 families in Glasgow	It is not reasonable to expect health visitors to robustly identify needs by the age of 4 months and that in very deprived populations some sort of ongoing universal service should be offered for at least the first year.	6/9<br><br>67%

**BRIDGE project**							
Enabling health and wellbeing among older people; Capitalising on resources in deprived areas through general practice	Wyke et al (2013) [55]	Consultation with experts	To develop a system through which general practices in deprived areas can identify older people in need and help them access resources and/or participate in activities known to help prevent or delay disablement and enhance wellbeing.	Mixed-methods (participative design and piloting).	3 Practices in Scotland	The likely 'active ingredients' of a general practice-based system included: a) Identification of a practice-based link worker; b) Active identification of people in need; c) Building relationships with community service providers; d) Providing older people with up-to-date information about services; e) Supporting older people to engage with services; f) Feedback and follow up.	Not peer reviewed
**Living Better**							
The Living Better Project: Addressing Mental health and Wellbeing in People Living with Long-term conditions	Maxwell et al. (2011) [56]	Targeted website search	To work with primary care health services to improve the way they address mental health and wellbeing, and in particular the detection and management of depression and anxiety	Qualitative study (focus groups, questionnaires and semi-structured interviews)	Five areas across Scotland, with a mix of urban/rural and deprived/more affluent areas	The project provides valuable insight into addressing mental health problems in multi-morbid patients. They specifically recommend support for managing financial concerns and making greater effort to target education at hard-to-reach individuals.	Not peer reviewed

			among people living with long-term conditions across Scotland.				
Homelessness outreach Service							
Benefits of GP care in outreach settings for people experiencing homelessness: A qualitative study	Hirst & Cuthill (2021) [57]	Database search	To explore PEHs' experiences of GP care in community outreach settings in UK; and to seek staff/volunteers' views on the strengths and weaknesses of GP community outreach services	Qualitative study (semi-structured interviews and focus groups)	3 sites across the UK, one of which is in Scotland	GP outreach services better enabled PEH to access medical care and staff/volunteers valued GP support to promote, and facilitate access to, healthcare services. Valued aspects of GP outreach were identified as comfortable, safe, and engendering a sense of belonging; convenient, opportunistic, and a one-stop shop; and being heard, having more time, and breaking down barriers.	7/10<br><br>70%
CARE Plus							
Exploring the utility of self-determination theory in complex interventions in multimorbidity: A qualitative analysis of	McCallum et al. (2021) [58]	Database search	To explore patients' experience of CARE Plus and whether self-determination theory is useful to	Qualitative study (semi-structured interviews)	14 participants	Participants valued the CARE Plus consultations irrespective of perceived improvements. Six participants reported changes in wellbeing that improved daily life, three	7/10<br><br>70%

patient experiences of the CARE Plus intervention			understand reported impacts.			reported slight improvement (not impacting daily life) and five no improvement.	
More time for complex consultations in a high-deprivation practice is associated with increased patient enablement	Mercer et al. (2007) [59]	Database search	To evaluate the effect of increasing consultation length on patient enablement in general practice in an area of extreme socioeconomic deprivation.	Non-randomised experimental design (longitudinal study with before and after design)	300 patient pre-intervention and 324 patients at follow-up at Keppoch Medical Centre in Glasgow.	GP stress in consultations was decreased after the introduction of longer consultations, and patient enablement was increased. GPs' views endorsed these findings, with more anticipatory and coordinated care being possible in the longer consultations.	9/9<br><br>100%
The development and optimisation of a primary care-based whole system complex intervention (CARE Plus) for patients with multimorbidity living in areas of high socioeconomic deprivation	Mercer et al. (2016) [60]	Database search	To develop and optimise a primary care-based complex intervention (CARE Plus) to enhance the quality of life of patients with multimorbidity in the deprived areas.	Qualitative study (co-design discussion groups, followed by piloting in two practices and further optimisation based on interviews)	32 participants- multimorbid patients from the deprived areas, voluntary organisations, general practitioners and practice nurses working in the deprived areas. Piloting in two practices and further optimisation based on interviews with 11 general practitioners, 2 practice nurses and 6 participating multimorbid patients.	Participants endorsed the need for longer consultations, relational continuity and a holistic approach. The pilot study led to changes including a revised care plan, the inclusion of mindfulness-based stress reduction techniques in the support of practitioners and patients, and the streamlining of the written self-management support material for patients.	7/10<br><br>70%

The CARE Plus study- a whole-system intervention to improve quality of life of primary care patients with multimorbidity in areas of high socioeconomic deprivation: Exploratory cluster randomised controlled trial and cost-utility analysis	Mercer et al. (2016) [61]	Database search	To evaluate a whole-system primary care-based complex intervention, called CARE Plus, to improve quality of life in multimorbid patients living in areas of very high deprivation	Cluster randomised controlled trial	152 patients participated (67/76 in each arm completed the 12-month assessment). Eight practices were involved	CARE Plus significantly improved one domain of well-being (negative well-being). Positive well-being, energy, and general well-being were not significantly influenced. The incremental cost in the CARE Plus group was £929 per participant with a gain in quality-adjusted life years of 0.076, resulting in a cost-effectiveness ratio of £12,224 per quality-adjusted life year gained.	9/13<br><br>69%
Govan SHIP							
The Govan SHIP Project (Social & Health Integration Partnership) Report and Evaluation	McGarry (2020) [62]	Targeted website search	Adopt a person-centred focus based on need, develop multi-disciplinary team working and challenge embedded silo approaches, identify opportunities to shift demand through better use of services	Qualitative. Ethnographic study	3 practices in one Health centre and 1 practice in another health centre in Govan, Glasgow	The project succeeded in reducing overall demand when comparing with a similar group of practices. Multi-disciplinary team working converted from initially bruising encounters into effective shared care and management of risk. Addressing unmet need formed the dominant use of additional GP time.	Not peer reviewed

			with colleagues working to the top of their licence, create capacity for GPs to increase their support for more complex patients, and better understand demand across health and care services at GP practice level.				
A Qualitative Evaluation of the Govan SHIP: A Social and Health Integration Partnership Project	Harris et al. (2017) [63]	Targeted website search	Explore the key components of the Govan SHIP model: linked social work (SW) and social care workers (SCWs), GP extra time and multidisciplinary team working (MDTs).	Qualitative study (unstructured and semi-structured interviews, and non-participant observation of MDT meetings)	10 unstructured interviews and 21 semi-structured interviews	MDT working, SW, SCW involvement and the additional time allocated to GPs worked in synergy to create an integrated model of working that shows promise for addressing the inverse care law.	Not peer reviewed
Deep End Report 29: GP use of additional time at Govan Health Centre as part of the SHIP project	Watt et al. (2016) [64]	Targeted website search	Summarises how GPs used their additional time	Cross-sectional study (a review of administrative data and a review of diaries)	15 GPs across 4 practices	GP partners reported 136 activities, including 76 extended consultations with the patient present and 14 sessions viewing 25 case records with the patient absent. Other activities	Not peer reviewed

						included correspondence, reports, contacts with professional colleagues and attendance at a range of meetings, including child protection case conferences.	
**Scottish Deep End Project**							
General practitioners at the Deep End: the experience and views of general practitioners working in the most severely deprived areas of Scotland	Watt (2012) [65]	Database search	To captures the experience and views of general practitioners (GPs) at the Deep End, comprising GPs serving the 100 most deprived practice populations in Scotland.	Case report/expert opinion piece	Scotland-wide	Argues that by increasing the volume, quality and consistency of care provided for individual patients, and harnessing the intrinsic strengths of general practice – including coverage, continuity, coordination, flexibility, long-term relationships and trust – general practices in very deprived areas can improve population health and narrow inequalities	6/6<br><br>100%
Connecting with general practice to improve public health: Report of the primary care observatory and Deep End projects	Watt (2011) [66]	Targeted website search	Describes two activities, the Primary Care Observatory (PCO) Project and the Deep End Project, with the common aim to	Mixed methods (cross-sectional studies and meeting reports)	Scotland	Practices serving areas of blanket deprivation were shown to have high levels of disease prevalence, a mismatch of manpower resource relative to need and	Not peer reviewed

			establish a better understanding of the contact and coverage of general practices with the populations they serve and the potential value of these contacts to the NHS in its attempts to improve health and narrow inequalities.			associated evidence of unmet need.	
Deep End Pioneer Scheme							
A change model for GPs serving deprived areas	Blane et al. (2017) [67]	Database search	The scheme involves the recruitment of younger GPs (or fellows), the retention of experienced GPs, and their joint engagement in strengthening the role of general practice as the natural hub of local health systems.	Case report	6 Deep End fellows at 6 practices in Glasgow	States that the Pioneer scheme is still at an early stage, but the host GPs involved already report a decrease in stress and renewed enthusiasm for their work. Patient benefits will take longer to demonstrate.	6/6<br>100%
The Deep End GP Pioneer Scheme: a qualitative evaluation	Dhanani & Blane (2022) [68]	Consultation with experts	To qualitatively evaluate the experiences of	Qualitative study (semi-structured interviews)	9 lead GPs and 10 GP fellows	Five main themes are presented: Recruitment to the Pioneer Scheme; Work	8/10<br>80%

			participating lead GPs and GP fellows.			motivation and satisfaction; Mitigating health inequalities; Retention and changes in work pattern; and Suggestions for the future. Key ingredients of the scheme were the additional clinical capacity, protected time and to share learning within and between practices. There was strong support for the Scheme as a mechanism to improve GP recruitment and retention.	
New models of Primary Care							
National Evaluation of New Models of Primary Care in Scotland	Mercer et al. (2019) [69]	Targeted website search	To identify tests-of-change, employ a case study approach to assess impact and to integrate findings.	Tests of change	204 projects were identified across Scotland. Only 10% related to health inequalities despite funding being dependent on needing to specifically consider impact on heath inequalities.	These were: welfare advice workers in NHS Tayside, musculoskeletal physiotherapy as part of Govan SHIP, Link Workers and House of Care framework in Lanarkshire and Ayrshire and Arran. 8 recommendations are made but these are not specific to the ICL.	Not peer reviewed
Local Health Care Cooperatives							

What impact did the creation of Local Health Care Co-operatives have on indicators of practice resources and activity?	McLean & Sutton (2008) [70]	Database search	To examine whether participation in LHCCs had any measurable impact on six indicators of practice resources and activity	Cross-sectional study	Scotland-wide	Despite having similar registered populations to participants, non-participants had lower levels of resources at the start of the period and this differential widened over time. There was little evidence that inequality between LHCC practices narrowed more than in the other two groups.	8/8<br><br>100%
Voluntary or compulsory health care reform? The case of primary care organisations in Scotland	Simoens & Scott (2005) [71]	Database search	To examine whether the voluntary nature of membership was likely to exacerbate or reduce inequalities in the provision of primary care services	Cross-sectional study	Scotland-wide	Results suggest that voluntary participation in these new primary care organisations may reduce rather than exacerbate inequalities in the provision of primary care.	5/8<br><br>63%
Training for Healthcare staff							
Community development in primary care: opportunities and challenges	Hogg & Hanley (2008) [72]	Database search	To explore primary health care professionals' views on community development, and to identify the opportunities and barriers associated	Qualitative study (small group discussion pre- and post-training)	16 participants took part in four pre-training group discussions, while 20 participated in the five post-training discussions, and 15 in the four follow-up ones	The study participants were confident that they already had most of the skills required for community development, but because of their discussions they became more aware of the opportunities to use the approach. They did not describe the course as	7/10<br><br>70%

			with using this approach in practice			having a major impact on their work	
NES Equality and Diversity Outcomes and Mainstreaming Priorities, 2017-2021: Progress report, April 2017-March 2019	NES (2019) [73]	Targeted website search	Outlines activities by NES to meet their equalities and diversity outcomes	Outline of Training	Scotland-wide	There are online training courses specific to health inequalities. They have produced guidance on employment of under-represented groups. They offer 3 GP health inequalities fellowships practicing in areas of deprivation. There are efforts to promote widening access to undergraduate medical and nursing education to recruit from areas of deprivation.	Not peer reviewed
Participatory Action Research							
Involving deprived communities in improving the quality of primary care services: does participatory action research work?	Cawston et al. (2007) [74]	Database search	To create participation by local people in evaluating the primary care services available in the area and to bring about change as a result of this process.	Qualitative study (participatory action research methods – questionnaire and focus groups)	72 residents took part in 11 focus groups. 372 residents completed the questionnaire. Conducted within one local health care cooperative in Glasgow	The intervention created participation but with modest impact on primary care delivery.	8/10<br><br>80%
Infrastructure							

Lochgelly Community Health and Wellbeing Centre Project Initial Agreement Document	Dobson & Connor (2019) dobson [75]	Targeted website search	Makes proposal for new community health centre in area of deprivation	Infrastructure proposal	Lochgelly	N/a	Not peer reviewed
Improving Services in the North East of Glasgow: North East HUB Health and Care Centre Full Business Case	Bailey (2021) [76]	Targeted website search	Makes proposal for new North East Hub in area of deprivation	Infrastructure Proposal	Glasgow	N/a	Not peer reviewed
Policy documents, analyses & recommendations							
Can primary care reduce inequalities in mental health?	Craig et al. (2009) [77]	Database search	To explore the contributions that primary care could make to reducing and preventing inequalities in mental health through policy, local strategy and practice	Mixed methods (Interpretive policy analysis framework of 9 health and social policy documents, observation of a mental health needs assessment process, and interviews)	21 frontline professionals from 14 different disciplines from Scotland	Policy documents demonstrated a disjointed picture of definitions and actions and lacked a clear overall interpretation of inequalities in health or inequalities in mental health. The mental health needs assessment did not incorporate discussion about inequalities in mental health.	4/7<br><br>57%
Workload and reward in the Quality and Outcomes	Guthrie et al. (2006) [78]	Database search	To examine the distribution of workload and	Cross-sectional study	903 GMS practices in Scotland	Payment is poorly related to workload in terms of the number of patients on the	6/8<br><br>75%

Framework of the 2004 general practice contract			payment in the clinical domains of the QOF and assess against the stated aim of helping tackle health inequalities.	(retrospective analysis)		disease register, with up to 44-fold variation in payment per patient on the disease register for practices delivering the same quality of care. Practices serving deprived populations are systematically penalised under the implemented payment system.	
Impact of the GP contract on inequalities associated with influenza immunisation: A retrospective population-database analysis	Norbury et al. (2011) [79]	Database search	To determine the impact of the 2004 UK General Medical Services contract on the overall uptake of, and socioeconomic inequalities associated with, influenza immunisation.	Cross-sectional study (Retrospective analysis)	15 GP practices in Scotland	Overall uptake rose significantly and differences between practices narrowed considerably. However, socioeconomic and age inequalities in influenza immunisation persisted in the first 3 years of the QOF.	8/8<br><br>100%
Challenges in implementing GP clusters in Scotland: a qualitative study comparing the views of senior primary care stakeholders in	Kidd Iet al. (2023) [80]	Database search (update)	To compare predicted challenges of cluster implementation in 2016 with reported challenges in 2021.	Qualitative study	12 national key stakeholders in primary care	In 2016 key stakeholders predicted a couple of challenges. In 2021 the progress of clusters was perceived as suboptimal. Concerns were raised about a cycle of non-progression whereby clusters become	7/10<br><br>70%

2016 with those in 2021						frustrated and burnout in the face of multiple system barriers impeding their work, leading to disengagement and further stagnation.	
Primary care transformation in Scotland: qualitative evaluation of the views of national senior stakeholders and cluster quality leads	Donaghy et al. (2023) [81]	Database search (update)	To explore progress in the implementation of the GP contract in Scotland in terms of the MDT and cluster working.	Qualitative study	Key national primary care stakeholders (PCSs) (n = 6) and cluster quality leads (CQLs) in clusters serving urban high deprivation areas (n = 4), urban mixed areas (n = 4), and remote and rural areas (n = 4)	Key PCSs and CQLs in different areas of Scotland report limited progress in primary care transformation, only partly related to the pandemic. There is a need for better workforce planning and support if the new GP contract is to succeed.	8/10<br><br>80%
Primary care transformation in Scotland: qualitative evaluation of the views of patients	Donaghy et al. (2024) [82]	Database search (update)	To explore patients' views on the changes in general practice in Scotland since the inception of the new contract	Qualitative study	30 patients (10 living in urban deprived areas, 10 living in urban affluent/mixed urban areas, and 10 living in remote and rural areas)	The patients were mainly unaware of the new GP contract. Although most were accepting of the increased role of MDT staff in general practice, face-to-face consultations with a known GP, together with more time and continuity of care were high priorities. These issues were especially concerning for patients with multiple complex problems,	8/10<br><br>80%

						particularly those from deprived areas.	
Primary care transformation in Scotland: a qualitative study of GPs' and multidisciplinary team members' views	Donaghy et al. (2024) [83]	Database search (update)	To explore practitioners' views on the expansion of MDT working in Scotland	Qualitative study	GPs and a range of MDT staff working in three different population settings in Scotland	There were many internal and external challenges to the expansion and implementation of integrated MDT working in primary care in Scotland. Challenges were most marked in deprived areas and in remote and rural settings.	8/10<br><br>80%
Patients' experiences of GP consultations following the introduction of the new GP contract in Scotland: a cross-sectional survey	Sweeney et al. (2024) [84]	Database search (update)	To determine the health characteristics and experiences of patients consulting GPs in deprived urban, affluent urban, and remote and rural areas of Scotland	Cross-sectional study	1053 participants of a random sample of adult patients from 12 practices who had consulted a GP within the previous 30 days was undertaken	The Scottish GP contract is an example of the global efforts to transform the delivery of primary care, with MDT expansion being a critical component, but there is a dearth of evidence reflecting patients' experiences of these efforts.	6/8<br><br>75%
The 2018 General Medical Services Contract in Scotland	Scottish Government (2018) [85]	Targeted website search	Outlines new GMS contract	Contract	Scotland	Funding is weighted by deprivation and age of population.	Not peer reviewed
Report of the Primary Care Health Inequalities Short-Life Working Group	Scottish Government (2022) [9]	Google search	Sets out recommendations for actions to be taken by primary care health	Recommendations for policy	Scotland	N/a	Not peer reviewed

			services to help reduce health inequalities and improve health equity in Scotland.				
National health and social care workforce plan: Part 3 – Improving workforce planning for primary care in Scotland	Scottish Government (2018) [6]	Targeted website search	Six long-term outcomes are described in this plan. It focuses on developing, building and expanding Multidisciplinary Teams (MDTs).	Policy	Scotland	N/a	Not peer reviewed
What can NHS Scotland do to prevent and reduce health inequalities? Proposals from General Practitioners at the Deep End	Alexander et al. (2013) [86]	Targeted website search	Outlines how NHS Scotland could and should address the inverse care law	Recommendations for policy	Scotland	N/a	Not peer reviewed
Progress Report on the 2022 Recommendations from the Primary Care Health Inequalities Short-Life Working	Scottish Government (2023) [87]	Google search (update)	To show the breadth of work that is progressing in primary care to address health inequalities, but also	Progress report	Scotland	Challenges are seen in the most deprived areas with a lower proportion of clinical staff. There were concerns about sustainability of CLW programme. Welfare advisers	Not peer reviewed

			highlights the significant challenges that remain			had a positive impact with a good financial gain	
GP Cluster Working Learning Cycle	Healthcare Improvement Scotland (2022) [88]	Google search (update)	To reflect on cluster working and discuss the next steps at both a local and national level. The findings from this learning cycle will inform future iterations of government policy for clusters.	Mixed-methods (learning cycle and interviews)	21 key stakeholders	Clusters were widely accepted as having great potential to improve the quality of care. However, implementation has not been fully supported or prioritised. They achieved a substantial cultural change as GPs are now coming together to share and discuss quality issues. The main barriers described were: time limitations, lack of support for clusters, lack of availability of meaningful data, and lack of clarity or different interpretations of the purpose, roles and responsibilities of clusters, HSCPs and NHS boards.	Not peer reviewed
Is Scotland's new GP contract addressing the inverse care law?	Mercer et al. (2023) [89]	Google search (update)	To investigate whether health inequalities have been addressed in	Cross-sectional study	Scotland	The high level of healthcare need in the poorest parts of Scotland, as indicated by the large inequalities in avoidable	5/7<br>71%

			general practice since the introduction of the contract			mortality, has not been matched by a supply of adequate levels of general practice clinical staff. There was a trend in the opposite direction, with higher numbers of general practice clinicians of all types concentrated in the practices serving the most affluent patients in Scotland.	
Collaborative improvement in Scottish GP clusters after the Quality and Outcomes Framework: a qualitative study	Huang et al. (2021) [90]	Google search (update)	To understand the organisation and perceived impact of GP clusters, including how they use quantitative data for improvement	Qualitative research	22 GP cluster professionals and other primary care improvers	Cluster development would benefit from more consistent training and support for cluster leads in small-group facilitation, leadership, QI expertise, data analytics. access and capacity. While GP clusters are operational, their impact is likely to be limited without further investment in developing capacity in these areas.	7/10<br><br>70%
Frameworks							
Improving together: A National Framework	Scottish Government (2017) [5]	Targeted website search	Outlines how GP clusters will work to	Framework	Scotland	N/a	Not peer reviewed

for Quality and GP Clusters in Scotland			guide quality improvement				
The role of Health and Social Care Partnerships in reducing health inequalities	NHS Health Scotland (2018) [91]	Targeted website search	outlines practical actions, along six main themes, as a way of considering health inequalities right from the start of developing plans and priorities. It also outlines key policies and drivers strengthening the role of HSCPs in reducing health inequalities	Framework	Scotland	N/a	Not peer reviewed
Focus on inequalities: a framework for action	Craig (2011) [92]	Targeted website search	To develop a framework to support Community Health (and Care) Partnerships to plan, monitor and evaluate action on health inequalities	Framework	Scotland	N/a	Not peer reviewed
National Monitoring and Evaluation	Scottish Government (2019) [8]	Targeted website search	Details the Primary Care Outcomes Framework. The	Framework	Scotland	N/a	Not peer reviewed

Strategy for Primary Care in Scotland			Framework provides a shared structure (in the form of a logic model) to articulate how they expect to realise the Primary Care Vision. To be used as a conceptual and practical evaluation tool				
Health Inequalities Strategies							
NHS Lothian: Health Inequalities Strategy	NHS Lothian (2014) [93]	Targeted website search	Outlines how NHS Lothian intends to respond to these inequalities and achieve greater equity in health for the Lothian population	Strategy document	Lothian health board	N/a	Not peer reviewed
Primary Care Improvement Plan	NHS Lanarkshire (2020) [94]	Targeted website search	Outlines how NHS Lanarkshire aims to target people living in deprived communities and outlines strategies to tackle health inequalities	Strategy document	Lanarkshire health board	N/a	Not peer reviewed

Action to address Health Inequalities in NHS Highland. Report for the NHS Highland Board.	Somerville (2014) [95]	Targeted website search	Summarises the main areas of work in progress in NHS Highland to reduce health inequalities and provide recommendations for future work to focus on	Strategy document	NHS Highland board	N/a	Not peer reviewed

Supplementary table S4: Included interventions, grouped by category.

Intervention (n=number of papers)	Description	Programme theory (I=Inputs, O=Outcomes, M=Mechanism)	Impact	Sustainability
Interventions that ***enhance financial or social support***				
Community Link Workers (n=19)	Practice-attached community practitioners offering non-clinical support, signposting, and health promotion.	Co-location of a new role (link workers) (I) to reduce health inequalities and promote wellbeing (O) through direct support or linking patients to community resources (M).	There was no difference in patient outcomes with the comparator group. Sub-group analysis demonstrated improved wellbeing for patients who saw the link worker on 3 or more occasions. There was correlation between link worker consultation rates and uptake of suggested resources. GPs, link workers and community organisations reported benefits – bridging between GP practices and organisations.	Has been sustained and rolled out nationally. Supported by GPs and patients. Staff are available to fill the role.
Embedded Welfare Advice (n=6)	Welfare advice workers attached to GP practices to support patients with	Embedding welfare advice workers (I) to increase financial gain	Increased the claiming of benefits among eligible patients. Financial gain demonstrated across	Has been sustained nationally, provides return on investment and staff are available.

	financial worries, benefits, debt and housing issues.	for patients (O) through advice and support with debt, housing, and financial issues (e.g. benefits applications) (M).	disability, child/maternity as well as debt management and task-shifting.	
Green Health Partnerships (n=1)	Social prescribing initiatives to promote and support use of green spaces for health improvement.	Creation of cross-sector partnerships (I) leading to promotion of health behaviours and improved mental health (O) though development of pathways for social prescribing using green spaces (M).	Green Health partnerships have been successfully created but no demonstrated benefit	To ensure sustainability, green health must be integrated into high-level strategic plans at national level. Currently green health interventions are delivered by third sector and volunteers.
Other Social Prescribing Initiatives (n=2)	Using non-clinical prescriptions for health promotion activities – such as exercise classes or social cafes.	Various mechanisms to promote social prescribing (I), improving physical and mental health (O) through support, enablement and improving access to community resources (M).	A complex landscape of roles and responsibilities has emerged across Scotland.	Funding structures for social prescribing were complicated and therefore vulnerable. Third sector organisations are also vulnerable to closure, restricting the options available for social prescribing roles to coordinate.
Interventions that ***target specific health conditions***				
Keep Well (n=7)	A national programme of improving cardiovascular anticipatory care in underserved populations.	Health checks for at risk individuals and creation of roles to enable access or provide outreach (I), to reduce mortality from	No improvement to mortality demonstrated. Various subjective benefits to staff and patients. Use of an outreach worker was believed to improve patient support and engagement.	Due to the high degree of uncertainty about the evidence supporting health checks the intervention was not sustained.

		cardiovascular disease (O) through changing how care is organised and delivered, focusing on clinical factors and pursuing empowerment and co-production (M).		
Blood Borne Virus Screening (n=3)	Offering screening to groups of patients in General Practice for blood borne viruses.	Offering blood borne virus screening to at risk populations through routine general practice (I), to reduce morbidity and mortality (O) through increased case identification and treatment (M).	Active screening – either new patients or during routine appointments – increases testing and identifies cases of Hepatitis C.	Provided funding was in place, screening of new or high-risk individuals could be sustainable. However, it is not clear if these interventions changed practice long-term.
Attached Alcohol Nurse Specialists (n=2)	Embedding nurse specialists into GP practices to perform targeted assessments and outreach of individuals with problem alcohol use.	Band 6 specialist addiction nurses attached to Deep End practices (I), to improve engagement with an underserved population (O), through a flexible service of in practice reviews, outreach and care planning (M).	Data collected suggests that A&E attendances increased in the time of the pilot. However, due to the small sample size and time course it was not statistically significant. The pilot was successful in achieving a working partnership between GPs and this 'specialist primary care service'. The flexible outreach approach was deemed beneficial.	If funding for the roles is provided there is no reason to suggest it would not be sustainable. Trained clinical staff are available.
Holistic interventions that ***target specific populations***				
Starting Well (n=5)	Combined an intensive health visitor schedule for	An intensive home visiting schedule	Subjective improvements in parent confidence, knowledge and skills. Complexities identified in the	Interviews found that there were complexities in

	families living in deprived areas with community development initiatives.	alongside community development (I) to improve child health and increase parental confidence and skills (O), through completion of a Family Health Plan, following a staged programme of topics, and the use of the Triple P parenting programme (M).	community-health component of the health visitor role.	implementation even with the pilot's high level of funding and input. Health visitors reported uncovering a large burden of need and escalating workload. The programme was not sustained.
Bridge Project (n=1)	Developed links between older patients in deprived areas and community-based resources to promote health and well-being.	Having a link worker, combined with building relationships with community service providers (I), to reduce social isolation, increase physical activity and enable improvements to health and wellbeing (O) though supporting older people to engage with services (M).	The practices were effective at identifying the right kinds of patients. Respondents were supportive of the intervention and voiced needs for support. Practices organised successful coffee mornings, taster walks and referrals to community organisations.	Interviewees voiced concerns around keeping up with the changing community resources, but felt that the connections should be sustainable at relatively low cost. However, there were concerns that enthusiasm would reduce over time. The primary issue for all practices was time – every aspect of the project took longer than expected.
Living Better (n=1)	Qualitative study of mental health issues in patients with long-term conditions, combined with training and resource development.	Integration of primary care with health, social and voluntary care providers, with increased training (I), to improve mental health and wellbeing for people living with	Mental health training for people with long-term conditions and for staff was successfully implemented, with local resources produced.	The projects were difficult to get up and running, and there were competing interests for time and resource.

		long term conditions (O) though coordinating HSCP staff and voluntary sector organisations, and through an assessment of patient needs and wants (M).		
Homelessness Outreach Services (n=1)	Outreach General Practice in homelessness centres to improve accessibility.	Integrating mainstream GP services into an outreach centre (I), to improve health outcomes and chronic disease management (O) by increasing engagement by persons experiencing homelessness (M).	Subjective improvement to patient accessing of healthcare reported in focus groups.	There is no specific reference to sustainability, however the outreach centres did rely on volunteers as well as medical staff.
Interventions that ***enhance generalist care***				
CARE Plus (n=4)	A complex intervention to improve consultations for patients with multi-morbidity in areas of deprivation.	Longer GP consultations, continuity and support for practitioners and patients (I), leading to improved Quality of Life and cost-effectiveness (O), via empathetic patient-centred care (M).	Increased time for patients in a deprived area was associated with improvements in quality of life and well-being. Cost effective. Patients and staff valued the intervention. Subjective improvements in wellbeing reported.	Staff shortages and high workload could reduce the ability for practices to engage with the intervention over a longer period of time. All 8 practices were able to complete the RCT and they achieved a high level of retention among patients.
Govan SHIP (n=3)	Integrated General Practice with Social Work, through MDT meetings,	Improved working with social work, alongside developing a patient-	Improved MDT working and GP additional capacity was used in a range of activities to the benefit of patients. However, did not have required critical	The intervention was not sustained due to concerns about costs. However, if, as

	additional GP capacity and co-location of Social Work.	centred focus and support for managing complex patients (I), which would reduce acute admissions and demand on secondary services, promote healthy living and improve chronic disease management (O) though increased GP capacity, employment of social care workers and structured MDT meetings (M).	mass to demonstrate effectiveness. Case reports of benefit.	demonstrated, the increased GP capacity and MDT working, increases efficiency of systems, reduces overall patient demand and demand on additional services, then this intervention is likely to be sustainable as it reduces burden.
Scottish Deep End Project (n=2)	A collaboration between academic and frontline GPs working in practices serving the 100 most deprived areas in Scotland.	Providing meetings, knowledge sharing and peer support for deep end GPs (I), leading to improving clinical care and staff retention (O) through support, education, advocacy and research activities (M).	Improved knowledge sharing and support, and advocated for improved services in deprived areas. Implemented the pioneer scheme. GPs reported less stress and supported the programme.	On-going since its inception, supported by GPs and has political backing.
Deep End Pioneer Scheme (n=2)	A fellowship scheme for early career GP fellows in Deep End practices, also provided protected time for experienced GPs to pursue service	Recruitment of early career GP fellows (I) to Deep End practices, with the aim of increasing recruitment of early career GPs and retention of older GPs	Qualitative improvements to confidence and knowledge. Supported by participants.	Only two cohorts of Deep End GP Pioneer Fellows were able to participate in the scheme, which ended as the COVID-19 pandemic struck. Due to enthusiasm for this approach,

	development and collaborative working.	(O). The GP fellows undertook a bespoke academic programme alongside clinical sessions and protected time to work on service development with an experienced GP (M).		there is support for relaunching this scheme.
New Models of Primary Care (n=1)	A range of primary care test-of-change projects, some of which aimed to address health inequalities.	Various test-of-change projects (I) which aimed to address health inequalities in areas of deprivation (O) through quality improvement or improving access (M).	Qualitative improvement to patient empowerment and resource utilisation for social prescribing.	Sustainability and spread relied heavily on both time and cost-saving strategies and ongoing support from practitioners.
Local Health Care Cooperatives (n=2)	Voluntary groups of GP practices within geographical areas to manage budgets, undertake commissioning and pursue quality improvement.	Creation of voluntary groups of GP practices (I) to pursue health care improvement (O) through patient and public involvement, working partnerships and collaboration between social work, acute sector and voluntary agencies (M).	Practices in deprived areas or practices with difficulty accessing local authority care homes were more likely to join an LHCC. It is not clear if joining LHCCs had a benefit.	Relies on political support and funding. They have since ceased to operate and been replaced by health and social care partnerships.
Training for Healthcare Staff (n=2)	A range of health inequalities-specific training available to healthcare staff.	Delivery of training to healthcare staff specific to health inequalities (I) which	Community development training course did not have a major impact on the work of participants. Other training not assessed formally.	No specific reference to sustainability, any training programmes would need to be repeated regularly.

		would mitigate health inequalities (O) by increasing awareness, knowledge and confidence (M).		
Participatory Action Research (n=1)	Involving a deprived population in shaping quality improvement of local primary care services.	Creation of a participatory action research group (I) leading to positive action in local primary care development (O) through engaging hard to reach individuals and involvement of residents of a deprived area in all aspects of study design, implementation and dissemination (M).	Participatory action in one community was possible, however there was modest impact on service delivery.	It is likely that such action would need to be regularly repeated in specific localities and therefore sustainability may be hard to achieve.
Infrastructure (n=2)	Examples of infrastructure improvements (e.g. premises) that were made in areas of deprivation.	Improving infrastructure in deprived areas (I), leading to improved provision of healthcare (O) through co-location of services and up-to-date facilities (M).	Proposed infrastructure only.	Long-term sustainability would be achieved with appropriate investment.

~~Supplementary file S1:~~ Further methods, search strategy and programme evaluation

Expanded methods:

Exclusion criteria were as follows; not Scotland; not General practice (e.g., other primary care providers such as dental or pharmacy); not targeted to deprived population; no intervention or policy described; conference proceedings, discussion papers, opinion pieces or editorials; paper is pre-2000; not written in English; and finally, if we were unable to locate or access the paper. We did not specify type of study, participants, or intervention, giving a wide scope in which to identify activities in Scotland.

Expanded search terms were chosen using guidance from standard syntax charts, and we employed a validated geographic filter [96,97]. Results were limited to humans, not animals, and to publication from the year 2000 onwards.

The grey literature review employed four search strategies; 1) grey literature databases; 2) search engine; 3) targeted websites; and 4) consultation with experts. Grey literature databases and relevant websites were identified using the Grey Matters guideline produced by Canada's Drug and Health Technology Agency [98], and through consultation with the project team. The Google search engine was employed for site-specific searches. Using the 'site:' search function, links were explored to the first 10 pages (100 results) by a single reviewer, and potentially relevant papers were downloaded. Websites were explored through browsing the site menu and employing internal search engines. Broad terms were used – General practice, Primary Care, inequality and inverse care law.

Search Syntax:

PubMed, MEDLINE

1. General practice [mh] OR Family Practice [mh] OR Community Medicine [mh] OR Social Medicine [mh] OR Comprehensive Health Care [mh] OR Primary Health Care [mh] OR General practitioners [mh]
2. General Practi* [tiab] OR GP [tiab] OR GPs [tiab] OR Primary care [tiab] OR Family practi* [tiab] OR Practitioner* [tiab] OR Communit* [tiab]
3. 1 OR 2
4. Scotland [mh]
5. (scotland* [tiab] OR scottish* [tiab] OR scotland* [ad] OR scottish* [ad])
6. (aberdeen [tiab] OR "aberdeen's" [tiab] OR dundee [tiab] OR "dundee's" [tiab] OR edinburgh [tiab] OR "edinburgh's" [tiab] OR glasgow [tiab] OR "glasgow's" [tiab] OR inverness [tiab] OR (perth not australia*) OR ("perth's" not australia*) OR stirling [tiab] OR "stirling's" [tiab] OR aberdeen [ad] OR "aberdeen's" [ad] OR dundee [ad] OR "dundee's" [ad] OR edinburgh [ad] OR "edinburgh's" [ad] OR glasgow [ad] OR "glasgow's" [ad] OR inverness [ad] OR (perth not australia*) OR ("perth's" not australia*) OR stirling [ad] OR "stirling's"[ad])

7. ("NHS Ayrshire and Arran" [tiab] OR "NHS Borders" [tiab] OR "NHS Dumfries and Galloway" [tiab] OR "NHS Fife" [tiab] OR "NHS Forth Valley" [tiab] OR "NHS Grampian" [tiab] OR "NHS Greater Glasgow and Clyde" [tiab] OR "NHS Highland" [tiab] OR "NHS Lanarkshire" [tiab] OR "NHS Lothian" [tiab] OR "NHS Orkney" [tiab] OR "NHS Shetland" [tiab] OR "NHS Tayside" [tiab] OR "NHS Western Isles" [tiab] OR "NHS Ayrshire and Arran" [ad] OR "NHS Borders" [ad] OR "NHS Dumfries and Galloway" [ad] OR "NHS Fife" [ad] OR "NHS Forth Valley" [ad] OR "NHS Grampian" [ad] OR "NHS Greater Glasgow and Clyde" [ad] OR "NHS Highland" [ad] OR "NHS Lanarkshire" [ad] OR "NHS Lothian" [ad] OR "NHS Orkney" [ad] OR "NHS Shetland" [ad] OR "NHS Tayside" [ad] OR "NHS Western Isles" [ad])
8. ((rural OR highland* OR island*) AND scot*[tiab])
9. (Lothian [tiab] OR lanarkshire [tiab] OR tayside [tiab] OR grampian [tiab] OR Orkney [tiab] OR shetland [tiab] OR Lothian [ad] OR lanarkshire [ad] OR tayside [ad] OR grampian [ad] OR Orkney [ad] OR shetland [ad])
10. 4 OR 5 OR 6 OR 7 OR 8 OR 9
11. (africa [mh] OR americas [mh] OR antarctic regions [mh] OR arctic regions [mh] OR asia [mh] OR oceania [mh]) not (great britain [mh] OR europe [mh])
12. 10 NOT 11
13. Health Policy [mh] OR Health Care Reform [mh] OR Health Care Quality, Access, and Evaluation [mh]
14. Policy [tiab] OR Policies [tiab] OR Intervention* [tiab] OR Program* [tiab] OR Project [tiab] OR Strategy [tiab] OR Strategies [tiab] OR Approach [tiab] OR Reform* [tiab] OR Initiative* [tiab] OR Pilot [tiab]
15. 13 OR 14
16. Delivery of Health care [mh] OR Health care reform [mh] OR Professional practice gaps [mh] OR Health Workforce [mh] OR Health care facilities, manpower and services [mh]
17. Suppl* [tiab] OR Fund* [tiab] OR Financ* [tiab] OR Payment* [tiab] OR Allocation* [tiab] OR "Global Sum" [tiab] OR Capitation [tiab] OR Money [tiab] OR Premise* [tiab] OR Surger* [tiab] OR Practice [tiab] OR Commission* [tiab] OR Access [tiab] OR Workforce [tiab] OR Staff [tiab] OR Doctor* [tiab] OR Nurs* [tiab] OR Pharmac* [tiab] OR prescrib* [tiab] OR Physio* [tiab] OR Service* [tiab] OR Organi?ation [tiab] OR Quality [tiab] OR Appointment* [tiab] OR Servic* [tiab] OR Deliver* [tiab] OR Consultation* [tiab] OR Train* [tiab]
18. 16 OR 17
19. Healthcare disparities [mh] OR Health Inequities [mh] OR Health Status Disparities [mh] OR Health Services Accessibility [mh] OR Health equity [mh] OR Universal health care [mh] OR Socioeconomic factors [mh] OR Social Determinants of Health [mh]
20. Equit* [tiab] OR Inequit* [tiab] OR Inequal* [tiab] OR Unequal [tiab] OR Gap [tiab] OR Gaps [tiab] OR Gradient* [tiab] OR Distribut* [tiab] OR Inverse [tiab] OR "Inverse Care Law" [tiab] OR Under doctored [tiab] OR Depriv* [tiab] OR Disadvantage* [tiab] OR SIMD [tiab] OR "Scottish Index of Multiple Deprivation" [tiab] OR Povert* [tiab] OR Impover* [tiab] OR Access [tiab] OR Econom* [tiab] OR Socioecon* [tiab] OR Shortage* [tiab] OR Poor* [tiab] OR Vulnerab* [tiab] OR Barrier* [tiab] OR Engagement* [tiab]
21. 19 OR 20
22. 3 AND 12 AND 15 AND 18 AND 21
23. Animals [mh] NOT humans [mh]
24. 22 NOT 23
25. Limit yr= 2000-current

Embase, Ovid

1. exp General practice/ OR exp General practitioner/ OR exp General practise registrar/ OR exp Primary medical care/ OR exp Social Medicine/
2. (General Practi* OR GP OR GPs OR Primary care OR Family practi* OR Practitioner* OR Communit*).mp.
3. 1 OR 2
4. exp Scotland/
5. (scotland* OR scottish*).ti,ab,jx,in,ad.
6. (aberdeen OR "aberdeen's" OR dundee OR "dundee's" OR edinburgh OR "edinburgh's" OR glasgow OR "glasgow's" OR inverness OR (perth not australia*) OR ("perth's" not australia*) OR stirling OR "stirling's").ti,ab,in,ad.

7. ("NHS Ayrshire and Arran" OR "NHS Borders" OR "NHS Dumfries and Galloway" OR "NHS Fife" OR "NHS Forth Valley" OR "NHS Grampian" OR "NHS Greater Glasgow and Clyde" OR "NHS Highland" OR "NHS Lanarkshire" OR "NHS Lothian" OR "NHS Orkney" OR "NHS Shetland" OR "NHS Tayside" OR "NHS Western Isles").ti,ab,in.
8. (lothian OR lanarkshire OR tayside OR grampian OR Orkney OR shetland).ti,ab,in.
9. ((rural or highland* or island*) and scot*).ti,ab,in,ad.
10. OR/4-9
11. (exp "arctic and antarctic"/ OR exp oceanic regions/ OR exp western hemisphere/ OR exp africa/ OR exp asia/ OR exp "australia and new zealand"/) not (exp united kingdom/ OR europe/)
12. 10 NOT 11
13. exp Health care policy/ OR exp health care planning/ OR exp Health program/
14. (Policy OR Policies OR Intervention* OR Program* OR Project OR Strategy OR Strategies OR Approach OR Reform* OR Initiative* OR Pilot).mp.
15. 13 OR 14
16. exp Health care delivery/ OR exp Health care quality/ OR exp health care utilization/ OR exp health workforce/ OR exp health care management/ OR exp health care organization/ OR exp health care personnel/ OR exp health care facility/
17. (Suppl* OR Fund* OR Financ* OR Payment* OR Allocation* OR "Global Sum" OR Capitation OR Money OR Premise* OR Surger* OR Practice OR Commission* OR Access OR Workforce OR Staff OR Doctor* OR Nurs* OR Pharmac* OR prescrib* OR Physio* OR Service* OR Organi?ation OR Quality OR Appointment* OR Servic* OR Deliver* OR Consultation* OR Train*).mp.
18. 16 OR 17
19. exp health care need/ OR exp health disparity/ OR exp health equity/ OR exp social determinants of health/ OR exp poverty/
20. (Equit* OR Inequit* OR Inequal* OR Unequal OR Gap OR Gaps OR Gradient* OR Distribut* OR Inverse OR "Inverse Care Law" OR Under doctored OR Depriv* OR Disadvantage* OR SIMD OR "Scottish Index of Multiple Deprivation" OR Povert* OR Impover* OR Access OR Econom* OR Socioecon* OR Shortage* OR Poor* OR Vulnerab* OR Barrier* OR Engagement*).mp.
21. 19 OR 20
22. 3 AND 12 AND 15 AND 18 AND 21
23. exp animals/ NOT exp humans/
24. 22 NOT 23
25. Limit year= 2000-current

CINAHL

1. (MH “Family Practice+”) OR (MH “Physicians, family+”) OR (MH “Primary Health Care+”) OR (MH “Community Medicine+”)
2. TX (General Practi* OR GP OR GPs OR Primary care OR Family practi* OR Practitioner* OR Communit*)
3. 1 OR 2
4. (MH “Scotland+”)
5. TX (scotland* OR scottish*)
6. TX (aberdeen OR "aberdeen's" OR dundee OR "dundee's" OR edinburgh OR "edinburgh's" OR glasgow OR "glasgow's" OR inverness OR (perth not australia*) OR ("perth's" not australia*) OR stirling OR "stirling's")
7. TX ("NHS Ayrshire and Arran" OR "NHS Borders" OR "NHS Dumfries and Galloway" OR "NHS Fife" OR "NHS Forth Valley" OR "NHS Grampian" OR "NHS Greater Glasgow and Clyde" OR "NHS Highland" OR "NHS Lanarkshire" OR "NHS Lothian" OR "NHS Orkney" OR "NHS Shetland" OR "NHS Tayside" OR "NHS Western Isles")
8. TX (lothian OR lanarkshire OR tayside OR grampian OR Orkney OR shetland)
9. TX ((rural or highland* or island*) and scot*)
10. 4 OR 5 OR 6 OR 7 OR 8 OR 9
11. (MH “Africa+”) OR (MH “America+”) OR (MH “Antarctic regions+”) OR (MH “Arctic regions+”) OR (MH “Asia+”) OR (MH “Australia+”) OR (MH “Indian Ocean Islands+”) OR (MH “Pacific Islands+”)
12. 10 NOT 11
13. (MH “Health Policy+”) OR (MH “Health Service Administration+”)

14. TX (Policy OR Policies OR Intervention* OR Program* OR Project OR Strategy OR Strategies OR Approach OR Reform* OR Initiative* OR Pilot)
15. 13 OR 14
16. (MH “Health Care reform+”) OR (MH “Health Resource Allocation+”) OR (MH “Health Resource Utilization+”) OR (MH “Health Manpower+”) OR (MH “Nursing Manpower+”) OR (MH “Health Personnel+”)
17. TX (Suppl* OR Fund* OR Financ* OR Payment* OR Allocation* OR "Global Sum" OR Capitation OR Money OR Premise* OR Surger* OR Practice OR Commission* OR Access OR Workforce OR Staff OR Doctor* OR Nurs* OR Pharmac* OR prescrib* OR Physio* OR Service* OR Organi?ation OR Quality OR Appointment* OR Servic* OR Deliver* OR Consultation* OR Train*)
18. 16 OR 17
19. (MH “Healthcare Disparities+”) OR (MH “Health Services Accessibility+”) OR (MH “Health Service Needs and Demands+”) OR (MH “Social Determinants of Health+”)
20. TX (Equit* OR Inequit* OR Inequal* OR Unequal OR Gap OR Gaps OR Gradient* OR Distribut* OR Inverse OR "Inverse Care Law" OR Under doctored OR Depriv* OR Disadvantage* OR SIMD OR "Scottish Index of Multiple Deprivation" OR Povert* OR Impover* OR Access OR Econom* OR Socioecon* OR Shortage* OR Poor* OR Vulnerab* OR Barrier* OR Engagement*)
21. 19 OR 20
22. 3 AND 12 AND 15 AND 18 AND 21
23. (MH “Animals+”) NOT (MH “Humans+”)
24. 22 NOT 23
25. Limit Year=2000-current

Web of Science

1. TS=(General practic*) OR TS=(Family Practic*) OR TS=(Community Medicine) OR TS=(Social Medicine) OR TS=(Comprehensive Health Care) OR TS=(Primary Health Care) OR TS=(General practitioners) OR TS=(GP) OR TS=(GPs) OR TS=(Primary care)
2. TS=(scotland*) OR TS=(scottish*) OR TS=(Aberdeen) OR TS=("aberdeen's") OR TS=(Dundee) OR TS=("dundee's") OR TS=(Edinburgh) OR TS=("edinburgh's") OR TS=(glasgow) OR TS=("glasgow's") OR TS=(inverness) OR TS=(perth not australia*) OR TS=("perth's" not australia*) OR TS=(stirling) OR TS=("stirling's") OR TS=("NHS Ayrshire and Arran") OR TS=("NHS Borders") OR TS=("NHS Dumfries and Galloway") OR TS=("NHS Fife") OR TS=("NHS Forth Valley") OR TS=("NHS Grampian") OR TS=("NHS Greater Glasgow and Clyde") OR TS=("NHS Highland") OR TS=("NHS Lanarkshire") OR TS=("NHS Lothian") OR TS=("NHS Orkney") OR TS=("NHS Shetland") OR TS=("NHS Tayside") OR TS=("NHS Western Isles") OR TS=(Lothian) OR TS=(Lanarkshire) OR TS=(tayside) OR TS=(Grampian) OR TS=(Orkney) OR TS=(shetland) OR TS=((rural or highland* or island*) and scot*)
3. TS=(Policy) OR TS=(Policies) OR TS=(Intervention*) OR TS=(Program*) OR TS=(Project) OR TS=(Strategy) OR TS=(Strategies) OR TS=(Approach) OR TS=(Reform*) OR TS=(Initiative*) OR TS=(Pilot) OR TS=(Health Policy) OR TS=(Health Care Reform) OR TS=(Health Care Quality, Access, and Evaluation) OR TS=(Health care policy) OR TS=(health care planning) OR TS=(Health program)
4. TS=(Suppl*) OR TS=(Fund*) OR TS=(Financ*) OR TS=(Payment*) OR TS=(Allocation*) OR TS=(“Global Sum”) OR TS=(Capitation) OR TS=(Money) OR TS=(Premise*) OR TS=(Surger*) OR TS=(Practice) OR TS=(Commission*) OR TS=(Access) OR TS=(Workforce) OR TS=(Staff) OR TS=(Doctor*) OR TS=(Nurs*) OR TS=(Pharmac*) OR TS=(prescrib*) OR TS=(Physio*) OR TS=(Service*) OR TS=(Organi?ation) OR TS=(Quality) OR TS=(Appointment*) OR TS=(Servic*) OR TS=(Deliver*) OR TS=(Consultation*) OR TS=(Train*)
5. TS=(Equit*) OR TS=(Inequit*) OR TS=(Inequal*) OR TS=(Unequal) OR TS=(Gap) OR TS=(Gaps) OR TS=(Gradient*) OR TS=(Distribut*) OR TS=(Inverse) OR TS=(“Inverse Care Law”) OR TS=(Under doctored) OR TS=(Depriv*) OR TS=(Disadvantage*) OR TS=(SIMD) OR TS=(“Scottish Index of Multiple Deprivation”) OR TS=(Povert*) OR TS=(Impover*) OR TS=(Access) OR TS=(Econom*) OR TS=(Socioecon*) OR TS=(Shortage*) OR TS=(Poor*) OR TS=(Vulnerab*) OR TS=(Barrier*) OR TS=(Engagement*) OR TS=(Healthcare disparities) OR TS=(Health Inequities) OR TS=(Health Status Disparities) OR TS=(Health Services Accessibility) OR TS=(Health equity) OR TS=(Universal health care) OR TS=(Socioeconomic factors) OR TS=(Social Determinants of Health)
6. 1 AND 2 AND 3 AND 4 AND 5
7. Limit year=2000-current

Example of SSPC programme evaluation.

Keep Well

National programme of anticipatory care in primary care settings. Launched in 2006 across five Scottish regions with high levels of deprivation, with the stated aim of contributing to a reduction in health inequalities in Scotland by providing health checks targeting those aged 45-64 years old at particular risk of preventable serious ill health, predominantly heart disease, and offering appropriate interventions, services and follow-up. Funding ended in 2017.

<u>Step 1: Programme theory and expected outcomes</u>

- What was the planned intervention and how did this build on previous work or knowledge?
  - The aim was to specifically target, reach and engage those who were not engaged with health services, and support them to undertake modification to identified CVD risk factors (smoking cessation, weight loss and statin therapy).
  - The evidence base for such a health check approach (targeted or otherwise) at the time of programme development was equivocal and where it was supportive was drawn from single interventions in a trial environment rather than effectiveness evidence from targeted health checks.
- How was the intervention expected to reduce health inequalities?
  - The relative divergence of improvement in cardiovascular disease (CVD) outcomes by socioeconomic status was of political concern. It was felt that a targeted CVD screening programme could increase the rate of improvement in the most deprived socioeconomic groups. This would contribute to a reduction in the inequalities in CVD mortality between the most and the least well off.
- What were the key components of the intervention?
  - Different components – and implemented differently across the country.
  - For instance, in SW Glasgow, two new roles were developed:
    - The role of the Health Case Manager (HCM) was to provide one to one support for those with multiple or complex needs, involving intensive support to encourage patients to take up their referrals to health and wellbeing services.
    - The Community Health Outreach Worker (CHOW) aimed to encourage patients who had not responded to invitations from the GP practice to attend Keep Well screening and support them to attend other services. Their

support was more limited, but involved a larger number of people than the HCM.

- What were the expected impacts at the start of the intervention?
  - Three theories of change were found to exist across the Boards:
    - Theory 1: Changing the way care is organised and delivered.
    - Theory 2: Empowerment and co-production.
    - Theory 3: Focusing on clinical risk factors.
  - In general, within each NHS Board, one of these theories appeared to have driven local planning for Keep Well more than the others. A number of outcomes for Keep Well were suggested during stakeholder interviews which were not part of the original programme theory and are not possible to explore with available data. These include improved relationships and trust between practitioners and patients, and increased self-efficacy.
- Was the intervention designed, developed or adapted to the specific context of the local area?
  - There were a range of adaptations. Each wave of Keep Well was accompanied by national guidance and annual performance reporting on completed health checks. NHS Health Scotland had a programme management role nationally which included provision of support to Boards in operationalising the guidance documents. However, as Keep Well was rolled out across Scotland, each of the waves was accompanied by slightly different guidance. In different areas, the programme’s theory came to be defined in a variety of ways and NHS Boards adapted the programme to local circumstances.
  - Keep Well implementation across Scotland was highly variable in its form, focus, delivery setting and expected outcomes. While there are advantages in local flexibility, the disadvantages include difficulties in evaluating impact and uncertainty about the evidence supporting specific local approaches.
- Were key stakeholders (such as healthcare staff, patients, carers living in deprived areas) involved in the co-design of the intervention?
  - Not clear.

Step 2: Impacts, learning, spread and sustainability

- What actual impacts did the intervention have in relation to the expected impacts?
  - No evidence of impact as a cardiovascular intervention (e.g. CVD mortality, hospitalisations).

    - Key evaluation finding (NHS GGC report) was extensive variation, at three levels:
        - Engaging the population subgroups at highest risk.
        - Changing the health literacy, risk factors and behaviour of those who engage.
        - Sustaining adherence to any changes after the Keep Well consultation.
- Did the intervention, and the expected impacts changed over time?
    - There were several 'waves' of Keep Well which each brought on new areas and/or general practices and had slightly different requirements: Wave 1 (2006), Wave 2 (2007), Well North (2008), Wave 3 (2009) and Wave 4 (2009). As the programme evolved it incorporated other population groups and initiatives. A process of 'mainstreaming' began in April 2012 with the aim of making targeted health checks part of 'normal, permanent practice' by 2014. In 2013, the Chief Medical Officer announced that central funding for Keep Well will cease in 2017.
- Were there any unintended (negative or positive) consequences?
    - The Keep Well programme encouraged innovation in the ways primary care sought to contact and engage deprived populations and those likely to be at high risk of CVD. In terms of collaboration between primary care and other services there is little evidence from local evaluation studies that this improved as a result of Keep Well. Despite this, interviewed stakeholders reported that Keep Well had improved working relationships between agencies and raised the profile and understanding of health inequalities locally.
    - The evaluation by O'Donnell et al identified 4 underlying tensions in the delivery of an anticipatory care approach through general practice:
        - General practice versus health improvement approaches;
        - Medical approaches versus wider social approaches;
        - Population-wide approach versus individual targeting;
        - Reactive versus anticipatory care.
- What was the key learning?
    - As above, the main NHS Health Scotland evaluation outlined three main lessons related to: 1) Problematic theory underling the intervention, 2) Variations in implementation, 3) Barriers to an effective assessment of impact.
    - Community Oriented Primary Care (COPC) clusters may offer opportunities to improve strategic linkage at all levels and provide more coherent programme support to local health improvement systems.

    - Customised models of anticipatory care are likely to be required for defined subpopulations, building on the successes of the South Asian Anticipatory Care (SAAC) and Carers' pilots.
- Was the intervention worthy of scaling up and spreading (implementation), and did this happen? If not, why not?
    - The intervention was scaled up for a number of years. However, the main NHS Health Scotland evaluation concluded that, due to the high degree of uncertainty of evidence supporting health checks, and where (as in Keep Well) the intervention does not lend itself to short-term process measures as valid proxies for the desired outcomes, a substantial programme such as Keep Well should be implemented in the context of a controlled trial, with comparison groups, considering options such as cluster randomisation or stepped wedge designs.
- Was the intervention sustainable (or likely to be sustainable) in the long-term?
    - As above. Keep Well was not sustained in the long-term. The available literature does not explicitly state the reasons for this, although the recommendations from the main NHS Health Scotland evaluation give us a strong idea.

References:

1. NHS Scotland. Delivering for Health. Edinburgh; 2005.

2. The Scottish Government. Renewing Scotland's Public Services: Priorities for reform in response to the Christie Commission. Edinburgh; 2011.

3. Scottish Government. A National Clinical Strategy for Scotland. Edinburgh; 2016 Feb.

4. Calderwood C. Chief Medical Officer's Annual Report 2014-15: Realistic Medicine. Edinburgh; 2016 Jan.

5. Scottish Government. Improving Together: A National Framework for Quality and GP Clusters in Scotland. Scottish Government. Edinburgh; 2017 Jan.

6. Scottish Government. National health and social care workforce plan: Part 3 – Improving workforce planning for primary care in Scotland. Edinburgh; 2018 Apr.

7. Scotland NHS. National Health and Social Care Workforce Plan Part 1 – a framework for improving. 2017;(June).

8. Scottish Government. National Monitoring and Evaluation Strategy for Primary Care in Scotland. Edinburgh; 2019 Mar.

9. Scottish Government. Report of the Primary Care Health Inequalities Short-Life Working Group. Edinburgh; 2022 Mar.

10. Chng NR, Hawkins K, Fitzpatrick B, O'Donnell CA, Mackenzie M, Wyke S, et al. Implementing social prescribing in primary care in areas of high socioeconomic deprivation: Process evaluation of the "Deep End" community Links Worker Programme. British Journal of General Practice. 2021 Dec 1;71(713):E912–20.

11. Hanlon P, Gray CM, Chng NR, Mercer SW. Does Self-Determination Theory help explain the impact of social prescribing? A qualitative analysis of patients' experiences of the Glasgow "Deep-End" Community Links Worker Intervention. Chronic Illn [Internet]. 2021 Sep 1 [cited 2023 Oct 27];17(3):173–88. Available from: https://pubmed.ncbi.nlm.nih.gov/31053038/

12. Mackenzie M, Skivington K, Fergie G. "The state They're in": Unpicking fantasy paradigms of health improvement interventions as tools for addressing health inequalities. Soc Sci Med. 2020 Jul 1;256:113047.

13. Mercer SW, Fitzpatrick B, Grant L, Chng NR, McConnachie A, Bakhshi A, et al. Effectiveness of community-links practitioners in areas of high socioeconomic deprivation. Ann Fam Med. 2019 Nov 1;17(6):518–25.

14. Skivington K, Smith M, Chng NR, Mackenzie M, Wyke S, Mercer SW. Delivering a primary care-based social prescribing initiative: A qualitative study of the benefits and challenges. British Journal of General Practice. 2018 Jul 1;68(672):e487–94.

15. Smith M, Skivington K. Community Links: Perspectives of community organisations on the Links Worker Programme pilot and on collaborative working with primary health care. Glasgow; 2015 Mar.

16. Scottish Government. Links Project Report: Developing the connections between General Practices and their communities. Edinburgh: Scottish Government; 2012 May.

17. O'Donnell CA, Cunningham Y, Wood K, Dare S, Saunders K, Thomas L, et al. Evaluation of New Models of Primary Care in Scotland: Ayrshire and Arran Case Study [Internet]. Glasgow; 2019 Jan. Available from: http://www.gla.ac.uk/departments/generalpracticeprimarycarehttp://www.facebook.com/gppcglasgow

18. Alliance. Link Worker Programme Record of Learning Series 2: Social Determinants in Primary Care. Glasgow; 2016 May.

19. Alliance. Developing a Culture of Health: The role of signposting and social prescribing in improving health and wellbeing. Glasgow; 2017.

20. Gourley C, Charlton M. Links Worker Programme, Record of Learning, Module: Context and Creation of the Programme. Glasgow; 2015 Apr.

21. Wyper G, Dryden R, Anderson J, Wason D. Monitoring and evaluation of primary care in Scotland: the baseline position. Edinburgh; 2020 Sep.

22. Public Health Scotland. Learning from the community link worker early adopters. Edinburgh; 2020 Jul.

23. VHS, SJ services. VHS Gold star exemplars. Third sector approaches to Community Link Working across Scotland. Edinburgh; 2017 Apr.

24. Alliance, RCGP. Improving Links in Primary Care: Project Report. Glasgow; 2014 Sep.

25. Alliance. Glasgow "Deep End" Links Worker Programme Practice Audit Questionnaire Evaluation. Glasgow; 2016 Apr.

26. Smith F. ESSENTIAL CONNECTIONS: Exploring the range and scope of community link worker programmes across Scotland. Edinburgh; 2023 Nov.

27. Mekina M, Conduit-Turner K. Community Link Worker Programme - Mental Health and Wellbeing Deep Dive. Glasgow; 2022 Nov.

28. MacAllister S, Moir E, Huc S, Collard B. NHS North Highland Community Link Worker Project: Year 1 Report. 2023.

29. Hoskins R, Smith L. Nurse-led welfare benefits screening in a General Practice located in a deprived area. Public Health. 2002 Jul;116(4):214–20.

30. Hoskins R, Tobin J, McMaster K, Quinn T. Roll-out of a nurse-led welfare benefits screening service throughout the largest Local Health Care Co-operative in Glasgow: An evaluation study. Public Health. 2005 Oct;119(10):853–61.

31. Sullivan F, Metsis K, Cunningham K. Evaluation of New Models of Primary Care in Scotland: Tayside Case Study. Glasgow; 2019 Jan.

32. Sinclair J. The Deep End Advice Worker Project: embedding an advice worker in general practice settings. Glasgow; 2017 Sep.

33. Egan J, Robison O. Integrating money advice workers into primary care settings: an evaluation. Glasgow; 2019 Jan.

34. Sinclair J. Building Connections: co-locating advice services in general practices and job centres [Internet]. Glasgow; 2017 Dec. Available from: www.gcph.co.uk

35. McHale S, Pearsons A, Neubeck L, Hanson CL. Green health partnerships in Scotland; pathways for social prescribing and physical activity referral. Int J Environ Res Public Health. 2020 Sep 2;17(18):1–13.

36. Fixsen A, Barrett S, Shimonovich M. Weathering the storm: A qualitative study of social prescribing in urban and rural Scotland during the COVID-19 pandemic. SAGE Open Med. 2021;9.

37. Fixsen A, Barrett S. Challenges and Approaches to Green Social Prescribing During and in the Aftermath of COVID-19: A Qualitative Study. Front Psychol. 2022 May 16;13.

38. Carver H, Douglas MJ, Tomlinson JEM. The outreach worker role in an anticipatory care programme: A valuable resource for linking and supporting. Public Health. 2012 Sep 1;126(SUPPL.1).

39. Carver H, Douglas MJ, Tomlinson JEM, Byrne C. “It’s just a way of approaching things now”: Staff perspectives of an anticipatory care programme in Edinburgh. Glob Health Promot. 2012 Jun;19(2):37–43.

40. O’Donnell CA, Mackenzie M, Reid M, Turner F, Clark J, Wang Y, et al. Delivering a national programme of anticipatory care in primary care: A qualitative study. British Journal of General Practice. 2012 Apr;62(597).

41. Sinclair A, Alexander HA. Using outreach to involve the hard-to-reach in a health check: What difference does it make? Public Health. 2012 Feb 1;126(2):87–95.

42. Scoular A. Evaluation of “Keep Well” programme in NHS Greater Glasgow & Clyde. Glasgow; 2012 May.

43. FMR Research. Exploration of the Community Health Outreach Worker and Health Case Manager roles. Glasgow; 2010 Mar.

44. NHS Health Scotland. The impact of Keep Well: An evaluation of the Keep Well programme from 2006 to 2012. Edinburgh; 2014.

45. Anderson EM, Mandeville RP, Hutchinson SJ, Cameron SO, Mills PR, Fox R, et al. Evaluation of a General Practice Based Hepatitis C Virus Screening Intervention. Scott Med J. 2009;54.

46. Bell J, Allerton L, Grant L, McLeman L, Tonna I, Okpo E. Learning lessons to improve blood borne virus testing in primary care in Scotland. Public Health. 2018 Jun 1;159:14–6.

47. Cullen BL, Hutchinson SJ, Cameron SO, Anderson E, Ahmed S, Spence E, et al. Identifying former injecting drug users infected with hepatitis C: An evaluation of a general practice-based case-finding intervention. J Public Health (Bangkok). 2012;34(1):14–23.

48. Williamson A, Craig M. Deep End Report 31 - Attached Alcohol Nurse Deep End Pilot (July 2015–2016): final report. Glasgow; 2016 Dec.

49. King A. Primary Care Alcohol Nurse Outreach Service (PCANOS): Full Evaluation. Glasgow; 2022 Oct.

50. Mackenzie M. Benefit or burden: Introducing paraprofessional support staff to health visiting teams: The case of Starting Well. Vol. 14, Health and Social Care in the Community. 2006. p. 523–31.

51. Mackenzie M. “Doing” public health and “making” public health practitioners: Putting policy into practice in “Starting Well.” Soc Sci Med. 2008 Sep;67(6):1028–37.

52. McIntosh J, Shute J. The process of health visiting and its contribution to parental support in the Starting Well demonstration project. Health Soc Care Community. 2007 Jan;15(1):77–85.

53. Ross MK, De Caestecker L, Sinclair M, Lakey T. The Starting Well Health Demonstration Project. Journal of Primary Prevention. 2005 May;26(3):205–20.

54. Wright CM, Jeffrey SK, Ross MK, Wallis L, Wood R. Targeting health visitor care: Lessons from Starting Well. Arch Dis Child. 2009 Jan;94(1):23–7.

55. Wyke S, Dow C, Watt G, O’Donnell C, Hendry A, Bowes A, et al. Enabling health and wellbeing among older people; Capitalising on resources in deprived areas through general practice. Glasgow; 2013.

56. Maxwell M, Donaghy E, Woodhouse A, Mercer S, Lawton K, Wallace R, et al. The Living Better Project: Addressing Mental health and Wellbeing in People Living with Long-term conditions. Edinburgh; 2011.

57. Hirst V, Cuthill F. Benefits of GP care in outreach settings for people experiencing homelessness: A qualitative study. British Journal of General Practice. 2021 Aug 1;71(709):E596–603.

58. McCallum M, Gray CM, Hanlon P, O’Brien R, Mercer SW. Exploring the utility of self-determination theory in complex interventions in multimorbidity: A qualitative analysis of patient experiences of the CARE Plus intervention. Chronic Illn. 2021 Dec 1;17(4):433–50.

59. Mercer SW, Fitzpatrick B, Gourlay G, Vojt G, McConnachie A, Watt GCM. More time for complex consultations in a high-deprivation practice is associated with increased patient enablement. Br J Gen Pract. 2007 Dec;57(545):960–6.

60. Mercer SW, O’brien R, Fitzpatrick B, Higgins M, Guthrie B, Watt G, et al. The development and optimisation of a primary care-based whole system complex intervention (CARE Plus) for patients with multimorbidity living in areas of high socioeconomic deprivation. Chronic Illn. 2016 Sep 1;12(3):165–81.

61. Mercer SW, Fitzpatrick B, Guthrie B, Fenwick E, Grieve E, Lawson K, et al. The CARE Plus study - a whole-system intervention to improve quality of life of primary care patients with multimorbidity in areas of high socioeconomic deprivation: Exploratory cluster randomised controlled trial and cost-utility analysis. BMC Med. 2016 Jun 22;14(1).

62. McGarry V. The Govan SHIP Project (Social & Health Integration Partnership) Report and Evaluation. Glasgow; 2020.

63. Harris FM, McGregor J, Maxwell M, Mercer S. A Qualitative Evaluation of the Govan SHIP: A Social and Health Integration Partnership Project. Glasgow; 2017 Jun.

64. Watt G, Montgomery J, Mullin A, Cameron N, Maguire S, McGarry V. Deep End Report 29: GP use of additional time at Govan Health Centre as part of the SHIP project. Glasgow; 2016 Jun.

65. Watt G. General practitioners at the Deep End: the experience and views of general practitioners working in the most severely deprived areas of Scotland. The Royal College of General Practitioners - Occasional Papers. 2012 Apr;40.

66. Watt G. Connecting with general practice to improve public health: Report of the primary care observatory and Deep End projects. Glasgow; 2011 Jul.

67. Blane DN, Sambale P, Williamson AE, Watt GCM. A change model for GPs serving deprived areas. Ann Fam Med. 2017 May 1;15(3):277.

68. Dhanani S, Blane DN. The Deep End GP Pioneer Scheme: a qualitative evaluation. Aust J Prim Health. 2023 Apr;29(2):155–64.

69. Mercer S, Gillies J, Noble-Jones R, Fitzpatrick B. National Evaluation of New Models of Primary Care in Scotland. Glasgow; 2019 Jan.

70. McLean G, Sutton M. What impact did the creation of Local Health Care Co-operatives have on indicators of practice resources and activity? BMC Health Serv Res. 2008 May 16;8(1):1–8.

71. Simoens S, Scott A. Voluntary or compulsory health care reform? The case of primary care organisations in Scotland. Health Policy. 2005;72(3):351–8.

72. Hogg R, Hanley J. Community development in primary care: opportunities and challenges. Community Practitioner. 2008 Feb;81(1):22.

73. NES. NES Equality and Diversity Outcomes and Mainstreaming Priorities, 2017-2021: Progress report, April 2017-March 2019. Edinburgh; 2019.

74. Cawston PG, Mercer SW, Barbour RS. Involving deprived communities in improving the quality of primary care services: Does participatory action research work? BMC Health Serv Res [Internet]. 2007 Jun 18 [cited 2023 Nov 2];7(1):1–9. Available from: https://bmchealthservres.biomedcentral.com/articles/10.1186/1472-6963-7-88

75. Dobson C, Connor N. Lochgelly Community Health and Wellbeing Centre Project Initial Agreement Document. Lochgelly: NHS Fife; 2019.

76. Bailey A. Improving Services in the North East of Glasgow: North East HUB Health and Care Centre Full Business Case. Glasgow: Glasgow City HSCP; 2021.

77. Craig PM, Hanlon P, Morrison JM. Can primary care reduce inequalities in mental health? Public Health. 2009;123(1):e57–61.

78. Guthrie B, McLean G, Sutton M. Workload and reward in the Quality and Outcomes Framework of the 2004 general practice contract. The British Journal of General Practice. 2006 Nov 11;56(532):836.

79. Norbury M, Fawkes N, Guthrie B. Impact of the GP contract on inequalities associated with influenza immunisation: A retrospective population-database analysis. British Journal of General Practice. 2011;61(588).

80. Kidd C, Donaghy E, Huang H, Noble-Jones R, Ogilvie S, McGregor J, et al. Challenges in implementing GP clusters in Scotland: a qualitative study comparing the views of senior primary care stakeholders in 2016 with those in 2021. BJGP Open. 2023 Jun;7(2):BJGPO.2022.0152.

81. Donaghy E, Huang H, Henderson D, Wang HH, Guthrie B, Thompson A, et al. Primary care transformation in Scotland: qualitative evaluation of the views of national senior stakeholders and cluster quality leads. British Journal of General Practice. 2023 Mar;73(728):e231–41.

82. Donaghy E, Sweeney K, Henderson D, Angus C, Cullen M, Hemphill M, et al. Primary care transformation in Scotland: a qualitative evaluation of the views of patients. British Journal of General Practice. 2024 Jan 16;BJGP.2023.0437.

83. Donaghy E, Huang H, Henderson D, Wang HH, Guthrie B, Mercer SW. Primary care transformation in Scotland: a qualitative study of GPs' and multidisciplinary team members' views. British Journal of General Practice. 2024 Jan;74(738):e1–8.

84. Sweeney KD, Donaghy E, Henderson D, Huang H, Wang HH, Thompson A, et al. Patients' experiences of GP consultations following the introduction of the new GP contract in Scotland: a cross-sectional survey. British Journal of General Practice. 2024 Feb;74(739):e63–70.

85. Scottish Government. The 2018 General Medical Services Contract in Scotland. Scottish Government. Edinburgh, Edinburgh: The Scottish Government; 2017. p. 46.

86. Alexander P, Budd J, Sambale P, Watt G. What can NHS Scotland do to prevent and reduce health inequalities? Proposals from General Practitioners at the Deep End. Glasgow; 2013 Mar.

87. Scottish Government. Progress Report on the 2022 Recommendations from the Primary Care Health Inequalities Short-Life Working Group. Edinburgh; 2023 Oct.

88. iHUB. GP Cluster Working Learning Cycle . Glasgow; 2022.

89. Mercer SW, Lunan C, Henderson D, Blane DN. Is Scotland's new GP contract addressing the inverse care law? Future Healthc J. 2023 Nov 30;10(3):287–90.

90. Huang H, Jefferson ER, Gotink M, Sinclair C, Mercer SW, Guthrie B. Collaborative improvement in Scottish GP clusters after the Quality and Outcomes Framework: A qualitative study. British Journal of General Practice. 2021;71(710):E719–27.

91. NHS Health Scotland. The role of Health and Social Care Partnerships in reducing health inequalities. Edinburgh; 2018.

92. Craig P. Focus on inequalities: a framework for action. Glasgow; 2011 Oct.

93. NHS Lothian. NHS Lothian: Health Inequalities Strategy. Edinburgh; 2014 Dec.

94. NHS Lanarkshire. Primary Care Improvement Plan. Bothwell; 2020.

95. Somerville M. Action to address Health Inequalities in NHS Highland. Report for the NHS Highland Board. Inverness; 2014 Aug.

96. Ayiku L, Levay P, Hudson T, Craven J, Barrett E, Finnegan A, et al. The medline UK filter: development and validation of a geographic search filter to retrieve research about the UK from OVID medline. Health Info Libr J. 2017 Sep 1;34(3):200–16.

97. Ayiku L, Levay P, Hudson T, Craven J, Finnegan A, Adams R, et al. The Embase UK filter: validation of a geographic search filter to retrieve research about the UK from OVID Embase. Health Info Libr J. 2019 Jun 1;36(2):121–33.

98. CADTH Research Information Services. Grey Matters: a practical tool for searching health-related grey literature. Ottawa; 2019 Apr.
